# Evaluating Precipitation Features and Rainfall Characteristics in a Multi‐Scale Modeling Framework

**DOI:** 10.1029/2019MS002007

**Published:** 2020-08-21

**Authors:** Jiun‐Dar Chern, Wei‐Kuo Tao, Stephen E. Lang, Xiaowen Li, Toshihisa Matsui

**Affiliations:** ^1^ Mesoscale Atmospheric Processes Laboratory NASA Goddard Space Flight Center Greenbelt MD USA; ^2^ Earth System Science Interdisciplinary Center University of Maryland College Park MD USA; ^3^ Science Systems and Applications Inc. Lanham MD USA; ^4^ GESTAR Morgan State University Baltimore MD USA

**Keywords:** Superparameterization, multi‐scale modeling framework, TRMM precipitation features, two‐dimensionality, cyclic boundary, tropical precipitation

## Abstract

Cloud and precipitation systems are simulated with a multi‐scale modeling framework (MMF) and compared over the Tropics and Subtropics against the Tropical Rainfall Measuring Mission (TRMM) Radar‐defined Precipitation Features (RPFs) product. A methodology, in close analogy to the TRMM RPFs, is developed to produce simulated precipitation features (PFs) from the output of the embedded two‐dimensional (2D) cloud‐resolving models (CRMs) within an MMF. Despite the limitations of 2D CRMs, the simulated population distribution, horizontal and vertical structure of PFs, and the geographical location and local rainfall contribution of mesoscale convective systems (MCSs) are in good agreement with the TRMM observations. However, some model discrepancies are found and can be identified and quantified within the PF distributions. Using model biases in relative population and rainfall contributions, PFs can be characterized into four size categories: small, medium to large, very large, and extremely large. Four different major mechanisms might account for the model biases in each different category: (1) the two‐dimensionality of the CRMs, (2) a positive convection‐wind‐evaporation feedback loop, (3) an artificial dynamic constraint in a bounded CRM domain with cyclic boundaries, and (4) the limited CRM domain size. The second and fourth mechanisms tend to contribute to the excessive tropical precipitation biases commonly found in most MMFs, whereas the other mechanisms reduce rainfall contributions from small and very large PFs. MMF sensitivity experiments with various CRM domain sizes and grid spacings showed that larger domains (higher resolutions) tend to shift PF populations toward larger (smaller) sizes.

## Key Points

- Many characteristics of simulated rain features are in reasonable agreement with satellite observations
- Four different major mechanisms might account for the rainfall and precipitation feature biases in the model
- Medium to large rain features contribute the most to the model tropical precipitation biases

## 1 Introduction

The multi‐scale modeling framework (MMF) or superparameterization (SP), which replaces traditional cloud parameterizations with two‐dimensional (2D) cloud‐resolving models (CRMs) within a host atmospheric general circulation model (GCM), has been developed over the past two decades (Cheng & Xu, 2011; Grabowski & Smolarkiewicz, 1999; Khairoutdinov & Randall, 2001; Randall et al., 2003; Tao et al., 2009; Wang et al., 2011, 2015). Conventional cloud parameterizations, using simple one‐dimensional plume models and closure assumptions to empirically represent sub‐grid cloud and convection processes in global models, have long been recognized as a major source of uncertainty in climate simulations. An MMF with its global cloud‐resolving (or permitting) capability provides a feasible approach to simulate explicitly convection systems and break the cloud parameterization deadlock (Randall, 2013; Randall et al., 2003). MMFs have been successfully applied to study a wide range of atmospheric phenomena (see Randall et al., 2016 for a review of some major applications). In general, MMFs produce simulations superior to their host GCMs in many physical processes, including the diurnal cycle (Khairoutdinov et al., 2005; Painemal et al., 2015; Pritchard & Somerville, 2009; Zhang et al., 2008; Zhao et al., 2017), the Madden‐Julian Oscillation (MJO) (Benedict & Randall, 2011; Khairoutdinov et al., 2008; Thayer‐Calder & Randall, 2009), mesoscale convective systems (Chen & Kirtman, 2018; Elliott et al., 2016; Marchand et al., 2009), low clouds (Cheng & Xu, 2011, 2013; Parishani et al., 2017; Wang et al., 2015; Xu & Cheng, 2013a, 2013b), temperature and moisture variability (Kahn et al., 2011), and extreme rainfall (Kooperman et al., 2016).

Despite their successes, MMFs also possess common systematic model biases, most notably excessive precipitation in the western Pacific Ocean, the tropical eastern Pacific, Bay of Bengal, and the western Indian Ocean. Several possible causes for the biases have been advanced in the literature such as the use of 2D CRMs with cyclic lateral boundary conditions, which prevents precipitation systems from direct propagation to a neighbor GCM column (Khairoutdinov et al., 2005), a positive convection‐wind‐evaporation feedback that fuels tropical convection by enhancing low‐level moisture convergence (Luo & Stephens, 2006; Tao & Chern, 2017), and momentum transport (Cheng & Xu, 2014). Because of the nonlinear couplings and feedbacks between the host GCM and the embedded CRMs, the physical causes of these biases are very challenging to isolate and quantify. The current study attempts to shed new light on possible physical mechanisms by analyzing different precipitation types by sizes and spatial structures. Model‐derived precipitation features (PFs) are analyzed to quantify the rainfall contribution and biases of simulated precipitation systems of various sizes in comparison with those observed during the Tropical Rainfall Measuring Mission (TRMM) (Simpson et al., 1996).

The TRMM precipitation radar (PR), the first of its kind in space, was in operation for nearly 17 years (1997–2014) to measure the horizontal and vertical distribution of rain from space. It was a cross‐track scanning radar, operated at Ku‐band (13 GHz), that surveyed a swath of 220 km at a horizontal resolution of 5.0 km (4.3‐km pre‐boost) at nadir and a vertical resolution of 250 m from the Earth's surface to 20 km in altitude. The PR together with passive visible, infrared, microwave, and lightning sensors on the TRMM satellite provided unprecedented and near continuous three‐dimensional (3D) measurements of precipitating systems in the Tropics and Subtropics. Due to the enormous amount of data collected by the TRMM satellite, many studies developed different approaches for grouping satellite pixels that satisfied certain criteria into specific features (Cecil et al., 2005; Cifelli et al., 2007; Matsui et al., 2016; Nesbitt et al., 2000; Nesbitt et al., 2006). Liu et al. (2008) further improved the TRMM precipitation feature (PF) data set by using TRMM PR echoes and near surface rain rate to characterize precipitation systems based on size and linked them with measurements from the TRMM Microwave Imager (TMI), the Visible and Infrared Scanner (VIRS), and the Lightning Imaging Sensor (LIS) to form a comprehensive multi‐sensor data set. This data set has been used to study the diurnal cycle of convection (Liu & Zipser, 2008), warm rain systems (Liu & Zipser, 2009), lightning characteristics (Liu et al., 2011), and latent heat release over the Tropics (Liu et al., 2015).

Many previous MMF studies focused mainly on large‐scale atmospheric phenomena using only mean fields at the GCM grid (Chern et al., 2016; Khairoutdinov et al., 2005; Randall et al., 2016; Tao et al., 2009; Tao & Chern, 2017), while other studies have included detailed analysis of cloud and precipitation characteristics using satellite simulators (Marchand & Ackerman, 2010; Matsui et al., 2016; Zhang et al., 2008; Zhao et al., 2017). In this study, detailed cloud and precipitation data sets from the embedded 2D CRMs are used to investigate the macrostructures and microstructures and statistics of cloud and rainfall from individual precipitation systems. A methodology, in close analogy to the TRMM radar‐defined PFs (RPFs) method (Liu et al., 2008), is developed to derive model‐simulated RPFs from the 2D CRMs. This data set is used to enhance the understanding of simulated cloud and precipitation characteristics such as horizontal and vertical structures, intensity, precipitation type, occurrence frequency, and rainfall contributions over the whole Tropics and Subtropics as well as the regions with excessive rain biases.

The main objectives of this study are to (1) evaluate the performance of the Goddard MMF (GMMF) against the TRMM observations, (2) improve the understanding of PFs and their characteristics in the Tropics and Subtropics, (3) identify and quantify possible causes of tropical rainfall biases in the GMMF, and (4) assess the impacts of CRM domain and grid spacing on precipitation characteristics. The paper has the following organization. Section 2 describes the GMMF, the Goddard Satellite Data Simulator Unit (G‐SDSU), the TRMM RPF validation data set, and a methodology for deriving the simulated RPFs from the 2D CRM output. In section 3, the results of the GMMF control experiment are presented. Section 4 shows the results of the sensitivity experiments assessing the impact of CRM grid spacing and domain size. Section 5 contains the discussion and conclusions.

## 2 Model, Methodology, and Observations

### 2.1 The Goddard Multi‐Scale Modeling Framework (GMMF)

The GMMF used in this study (hereinafter referred as the GMMF v3.0) is a progressive revision of the GMMF v2.0, which was documented and evaluated against CloudSat, CALIPSO, and other satellite data in Chern et al. (2016). Briefly, the GMMF is based on embedding a 2D cloud‐resolving Goddard Cumulus Ensemble model (GCE, Tao & Simpson, 1993; Tao et al., 2014) into each column of the Goddard Earth Observing System (GEOS) global atmospheric model to replace standard GEOS parameterizations for sub‐grid convection, large‐scale moist processes, turbulence, and radiation. The GEOS with a finite‐volume dynamic core was run at 2° × 2.5° (latitude × longitude) horizontal grid spacing with 48 vertical layers stretching from the surface to 0.4 hPa on a terrain‐following hybrid pressure coordinate with 17 layers below 700 hPa to improve resolution in the lower atmosphere. All embedded GCEs used cyclic lateral boundaries and 44 vertical layers collocated initially with the GEOS grid layers. They had four fewer vertical layers to ensure their model top height was lower than that of GEOS due to the different vertical coordinates (height in GCE vs. hybrid pressure in GEOS) used. The GEOS‐GCE coupling time was 1 hr, the same as the GEOS model time step. In this study, a series of GMMF simulations (Table 1) were carried out with different combinations of GCE grid spacings (i.e., 1, 2, and 4 km) and domain sizes (128, 256, and 512 km). The 2D GCE domains in all GMMF experiments are aligned in an east‐west direction in this study. Khairoutdinov et al. (2008) showed that their MMF results are, to some extent, orientation dependent. The representation of convective momentum transport (CMT) in an MMF has been a long‐standing challenge due to the 2D nature of the embedded CRMs. Recently, Cheng and Xu (2014) and Tulich (2015) have included CMT in their MMFs. In this study, the GMMF is like other traditional MMFs in only considering the thermodynamic feedback.

**Table 1:** Experiment Name and Model Configuration for Five GMMF Simulations

EXP names	CRM grid columns	CRM grid spacing	CRM domain size	Model time step
NX256_1KM (control)	256	1 km	256 km	3 seconds
NX128_2KM	128	2 km	256 km	6 seconds
NX64_4KM	64	4 km	256 km	12 seconds
NX32_4KM	32	4 km	128 km	12 seconds
NX128_4KM	128	4 km	512 km	12 seconds

The Goddard one‐moment, four‐class ice (4ICE) bulk microphysical scheme (Lang et al., 2014; Tao et al., 2016) having six prognostic condensates (cloud water, rain, cloud ice, snow, graupel, and hail) was used in this study. The long‐wave (Chou et al., 1999) and shortwave radiation (Chou et al., 2002) schemes are the same as in GMMF v2.0; however, the optical properties of all major (water vapor, CO_2_, O_2_) and most of the minor trace gases (O_3_, N_2_O, CH_4_, CFCs) are updated to the HITRAN 2012 molecular spectroscopic data set (Rothman et al., 2013). The radiation schemes are called within the GCE to improve cloud‐radiation interaction at the natural temporal and spatial resolution of the CRM. Lastly, the air‐sea surface flux scheme of GEOS (Large & Pond, 1981, 1982) was replaced with Tropical Ocean‐Global Atmosphere Coupled Ocean‐Atmosphere Response Experiment (TOGA COARE) version 4 flux scheme (Edson et al., 2013; Fairall et al., 2003, 2011). The scheme increases surface drag over high wind regions based on in situ observations from more recent field campaigns. In this study, the TOGA COARE flux scheme is called from the GCM, and the hourly mean surface fluxes are applied uniformly within the GCE.

### 2.2 The Goddard Satellite Data Simulator Unit (G‐SDSU)

The G‐SDSU is an end‐to‐end multi‐satellite simulator unit with five simulators at present, including a passive visible‐IR simulator, a passive microwave simulator, a broadband simulator, a radar simulator, and a LiDAR simulator. It has been developed at NASA GSFC in collaboration with other institutions (Masunaga et al., 2010; Matsui et al., 2014) to support various NASA satellite missions (Matsui et al., 2013). The G‐SDSU converts the simulated thermodynamics and liquid/ice condensate profiles from a mesoscale or cloud‐scale model into satellite‐equivalent Level‐1 (L1) measurements such as radiance/brightness temperature or backscatter signals at model resolution. Then, the signals are convolved in the sensor field‐of‐view based on the antenna gain function (Matsui et al., 2013). The G‐SDSU has been coupled with various NASA high‐resolution model output, such as the GCE, the GMMF, the NASA‐Unified Weather Research and Forecasting (NU‐WRF) model, and GEOS version 5 (GEOS5). There are many microphysics options, including one‐ and two‐moment bulk schemes and spectral‐bin microphysics. Particle size distributions (PSDs) and various hydrometeor classes in these schemes are consistently treated among different simulators in the G‐SDSU.

Matsui et al. (2016) successfully coupled output from the GMMF and the Nonhydrostatic Icosahedral Cloud Atmospheric Model (NICAM) with the G‐SDSU to evaluate the land‐sea contrast in tropical convection versus TRMM measurements using the TRMM Triple‐Sensor Three‐Step Evaluation Framework (T3EF, Matsui et al., 2009). In this study, the G‐SDSU is used to convert 3‐hourly instantaneous GMMF v3 output into TRMM satellite measurable signals such as radiance/brightness temperature and radar reflectivity to enable the production of a Level‐2 simulated RPF data set.

### 2.3 Methodology and Validation Data Set

#### 2.3.1 Validation Data Set

The validation data set used in this study is the TRMM orbital Level‐2 Radar‐defined Precipitation Features (RPFs) product, one of several PF products in the comprehensive TRMM precipitation and cloud feature database (Liu et al., 2008), which was reprocessed in 2012 based on TRMM version 7 products. RPFs are first identified by grouping the contiguous pixels with near‐surface precipitation based on TRMM 2A25 PR rainfall retrievals (Iguchi et al., 2000, 2009). Then, their general information such as time, location, size (total pixel number), volumetric rain (km^2^ mm/hr), and stratiform and convective pixel numbers from TRMM 2A23 rainfall categorizations (Awaka et al., 1997, 2009; Steiner et al., 1995) are linked together. As a multi‐sensor data set, it also includes parameters such as maximum 20, 30, and 40 dBZ echo heights and vertical profile of maximum reflectivity from the TRMM PR, minimum and area of 85‐GHz polarization‐corrected temperature (PCT; Spencer et al., 1989) from the TMI, minimum brightness temperature (T_B11_) area from the VIRS, and lightning‐flash counts from the LIS (details are given in Liu et al., 2008).

Observed organized precipitation systems may appear as linear or nonlinear shapes under different environments (e.g., Johnson et al., 2005; LeMone et al., 1998; Liu & Zipser, 2013; Xu & Rutledge, 2015). Liu et al. (2008) applied an ellipse‐fitting technique (Nesbitt et al., 2006) on the rainfall area of each observed PF in the TRMM PR swath data. The aspect ratio, the major and minor axes, and the orientation angle of the major axis of the best‐fit ellipses are recorded in the TRMM RPF data set used in this study. Two examples of the PR swath image with best‐fit ellipses of PFs were shown in Figure 1 of Nesbitt et al. (2006). Ellipses of PFs with a variety of size, aspect ratio, and orientation may coexist within the same satellite scene. To evaluate the 1‐year (2007) simulations from the GMMF, the TRMM RPF data set for 2007 is used in this study.

#### 2.3.2 Methodology

The TRMM RPF database is mainly based on 2A25 PR rainfall retrievals (Iguchi et al., 2000) and 2A23 rainfall type categorizations (Awaka et al., 1997; Steiner et al., 1995). In the 2A25 algorithm, the TRMM PR‐measured radar reflectivity (Z_m_) is first corrected for rain attenuation using a hybrid of the Hitschfeld‐Bordan formulation (Hitschfeld & Bordan, 1954) and the surface reference method (Meneghini et al., 2000, 2004). Then, the vertical rain (R) profile is calculated from this attenuation‐corrected reflectivity (Ze) profile using an appropriate Ze‐R power‐law relationship (Iguchi & Meneghini, 1994) with parameters based on rain type, phase state, storm height, existence of a bright band, freezing height, air temperature and density, and so on. Since TRMM PR echoes near the ground are contaminated by surface clutter, the near‐surface rainfall rate is estimated at the lowest point free from surface clutter, typically 500–2,000 m above the ground depending on the radar scanning angle and the terrain height. Due to the PR sensitivity of ~17 dBZ, there are some limitations in retrieving light rainfall rates below ~0.5 mm hr^ − 1^. The PR‐observed rainfall is further categorized in 2A23 into three types: stratiform, convective, and other using two different methods, a vertical profile method (V‐method) based on the detection of a bright band (Awaka et al., 1997) and a horizontal pattern method (H‐method) based on the intensity and horizontal gradient of radar reflectivity (Steiner et al., 1995).

In this study, a methodology in close analogy to the TRMM RPFs is developed to produce simulated RPFs from the output of the embedded 2D GCEs within the GMMF. First, the G‐SDSU is used to convert simulated profiles of atmospheric temperature, moisture, and liquid/ice condensates into radar reflectivity signals at Ku‐band (13 GHz) frequency. Then, the signals are corrected for the rain attenuation and averaged to the field‐of‐view of the TRMM PR (~4.3 km in size) based on the antenna gain function (Matsui et al., 2013). Due to the complexity of the TRMM Ze‐R power‐law relationship, the G‐SDSU generates only radar reflectivity profiles without the rain profiles. To consider the effects of TRMM PR detection limits and surface clutter on the rain/no‐rain flag, a model rain pixel is defined as a grid point having a surface rain rate greater than 0.4 mm hr^ − 1^ (the approximate minimum values in the 2A25 data set) and radar reflectivity greater than 17 dBZ at 500 m above the ground. The model PFs are then defined by grouping the contiguous 2D rain pixels. The G‐SDSU has an option for considering the radar reflectivity of melting ice particles (i.e., bright band). However, this option was turned off because the simple one‐moment bulk microphysical scheme used in this study cannot provide the necessary information to generate a realistic melting/riming fraction for the G‐SDSU. As a result, the model stratiform and convective rainfall categorizations are only based on the H‐method (Awaka et al., 1997).

#### 2.3.3 Dimensionality Consideration

**Figure 1:**
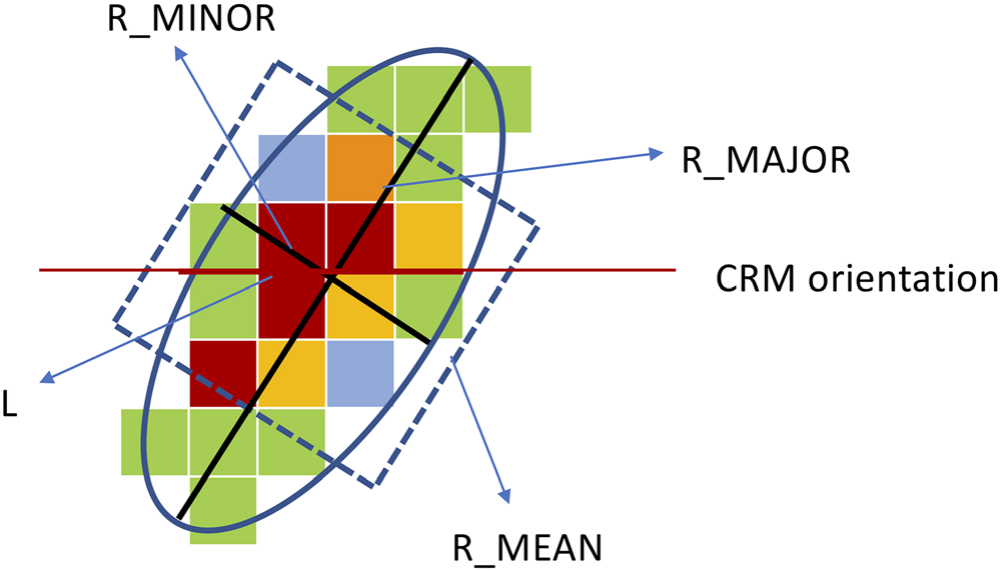
Schematic diagram illustrating the relationship among the four length scales (R_MINOR, R_MAJOR, R_MEAN, and L). The ellipse represents the best fit for a precipitation feature with rain pixels shown in color. The square has the same area as the rain feature.

Although PFs are defined by grouping contiguous rain pixels in both the TRMM and simulated RPFs, TRMM RPFs are usually categorized by their 2D rain area while simulated RPFs from the 2D CRMs are sorted by their one‐dimensional length (L). The square root of the rainfall area (R_MEAN) has been used in Li et al. (2018) to characterize the horizontal dimension of observed PFs. For PFs with a nearly circular shape, the simulated length scale L can be directly compared with the observed mean length scale R_MEAN. However, for PFs with an elliptic shape or squall lines, the east‐west orientation of the 2D GCE and precipitation systems can occur at an angle. The length scale L can represent different cross section lengths depending on the orientation of simulated PFs, which is unknown in a 2D CRM. As a result, it is impossible to make direct quantitative comparisons of the size of PFs between the TRMM and simulated RPFs. Fortunately, the TRMM RPF data set also provides the minor axis (R_MINOR) and major axis (R_MAJOR) of the best‐fit ellipses of observed PFs. Thus, TRMM RPFs associated with three observed length scales (R_MINOR, R_MEAN, and R_MAJOR) will all be compared with the simulated RPFs to estimate the range of uncertainty due to the dilemma in defining the PF size (L). Figure 1 shows a schematic diagram illustrating the relationship among these four length scales.

The observed volumetric rainfall of each PF, which in nature is 3D, is the sum of rainfall over its area; however, for a simulated PF in 2D, the volumetric rainfall is only the sum along its length. To compare with the model results, the 2D volumetric rainfall along all three observed axes (R_MAJOR, R_MINOR, and R_MEAN) is computed. This 2D rainfall contribution of PFs is used throughout this study except in the discussion of the rainfall contribution of MCSs, where both 2D and 3D contributions are used (see section 3.3). The 3D volumetric rainfall of a PF is proportional to the square of its length. As a result, large PFs such as MCSs will have a higher percentage of rainfall contribution in 3D than in 2D. For the sole purpose of comparing MCSs rainfall contribution from this study with TRMM observational studies in the literature, a simulated 3D rainfall contribution diagnostic is needed. Since rainfall information is only available along the orientation of the 2D CRM, an ad hoc assumption about the area of a simulated PF is required for this diagnostic. It is assumed each simulated PF has a circular shape with the same mean rain rate in this study. Other shapes can also be used as long as this diagnostic is proportional to L^2^ instead of L. It is expected that some uncertainties might arise due to this assumption.

## 3 GMMF Control Experiment Results

**Figure 2:**
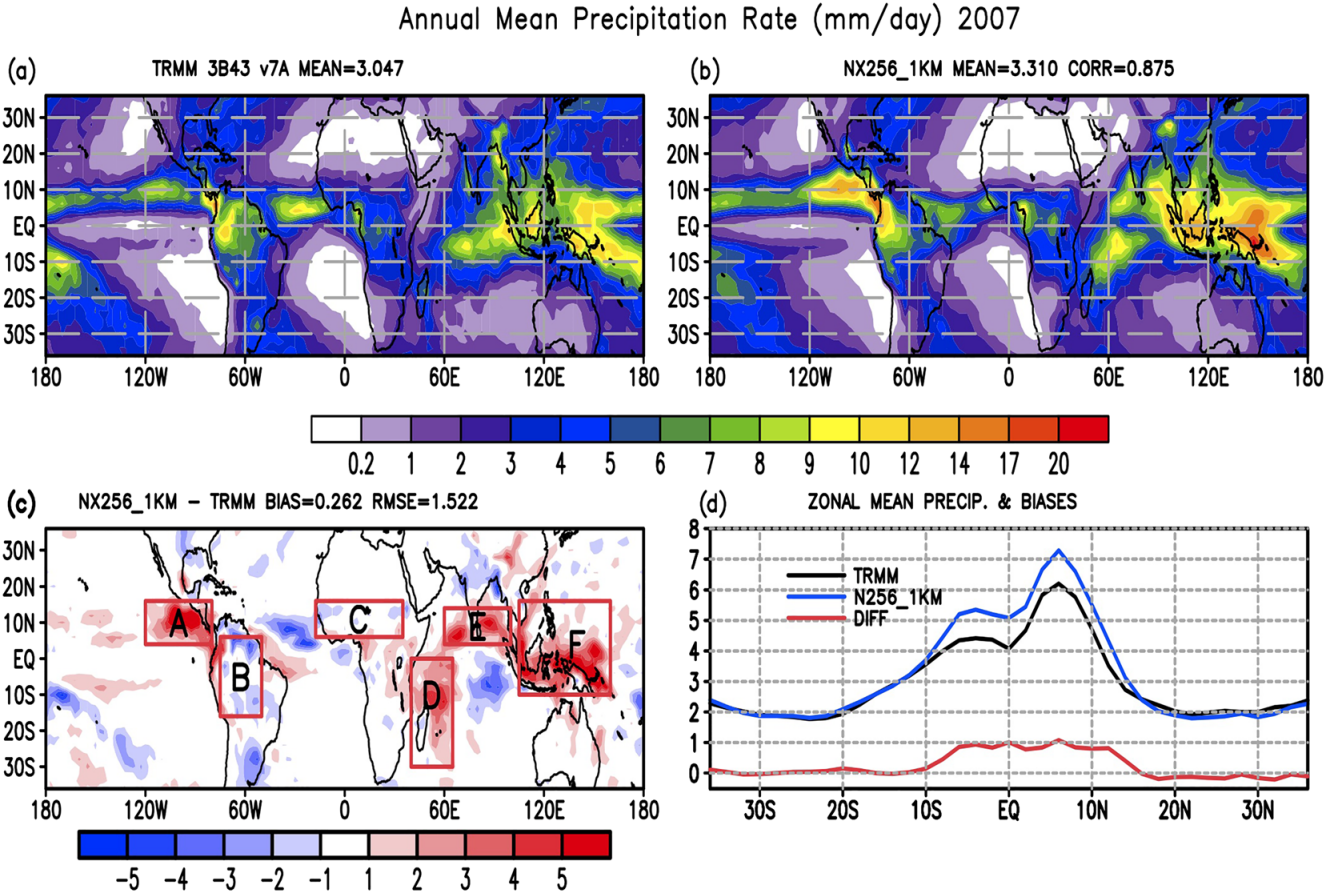
Annual mean precipitation rates (mm/d) from (a) TRMM 3B43 observations, (b) the GMMF control simulation, (c) their differences, and (d) their zonal mean and zonal mean difference for 2007.

Table 1 lists the configuration of all GMMF control and sensitivity experiments presented in this paper. The GMMF control experiment (NX256_1KM) has 256 GCE grid columns, 1‐km horizontal grid spacing, and a GCE model time step of 3 seconds. A series of sensitivity experiments are also carried out to study the effects of CRM domain size (NX32_4KM, NX64_4KM, NX128_4KM) and grid spacing (NX128_2KM, NX64_4KM) on simulated PFs. All experiments are integrated from 1 December 2006 to 31 December 2007 using initial atmospheric conditions from the ECMWF ERA‐Interim reanalysis (Dee et al., 2011) at 0000 UTC 1 December 2006. The first month is considered as spin‐up, so only results from 2007 are used in this study. Sea surface temperatures (SSTs) were prescribed using NOAA optimal interpolation (OI) weekly SSTs (Reynolds et al., 2007). Figure 2 shows the geographical distributions of simulated annual mean precipitation from the control simulation, along with the corresponding observations from the TRMM 3B43 product (Huffman et al., 2007, 2010). In general, given the observed SST forcing, the observed annual mean precipitation patterns in the Tropics and Subtropics can be realistically simulated by the GMMF in a “free run” mode. However, there is a systematic overestimation of precipitation in several oceanic regions such as the western and northern Indian Ocean, the tropical warm pool and the tropical western Pacific, and the tropical eastern Pacific, while precipitation in the Amazon and the central Indian Ocean is underestimated. These positive biases in the tropics are quite common among different MMFs (Tao et al., 2009; Tao & Chern, 2017). The zonal mean shows an overestimation of rainfall in the Tropics from 10°S to 15°N.

### 3.1 Population and Rainfall Contributions of PFs in the Tropics and Subtropics

To compare with TRMM RPFs and other sensitivity experiments, the precipitation in the control run is averaged from a 1‐km to a 4‐km grid, and the radar reflectivity signals are convolved into the TRMM PR field‐of‐view (~4.3 km) in the G‐SDSU. The embedded CRMs with a typical grid spacing of 4 km cannot resolve waves with a wavelength less than or equal to two model grids (8 km). To avoid this problem as well as possible data noise in the TRMM PR measurements having only a few pixels, PFs with a size less than 12 km are ignored in this study. All distribution functions of rain characteristics as a function of system size correspond to PFs ≥ 12 km. Table 2 shows the numbers, number percentages, and rainfall contribution of observed and simulated PFs over the Tropics and Subtropics (36°S–36°N) for different PF size ranges. The observations have about 6.59 million PFs while the control experiment has about 10.6 million PFs derived from the 3‐hourly CRM output for 2007. Most of the observed PFs are small; PFs having a size ≥ 12 km only account for about 11.5%, 14.6%, and 32.2% of the total PFs for R_MINOR, R_MEAN, and R_MAJOR, respectively. R_MAJOR has a larger percentage as it tends to classify small PFs to bigger sizes. All GMMF experiments underestimate the occurrence of small PFs with a size   <   12 km. Due to their two‐dimensionality, the CRMs have one less degree of freedom to support many concurrent small PFs versus the observations.

**Table 2:** Numbers, Number Percentages, and Rainfall Contribution (%) of Observed and Simulated Precipitation Features Over the Tropics and Subtropics (36°S–36°N) for Different PF Size Ranges

Name	**All** PFs number (PFs ≥ 4 km)	**Total** (PFs ≥ 12 km)	**Small** (12 ≤ PFs < 20 km)	**Medium to Large** (20 ≤ PFs < 90 km)	**Very Large** (90 ≤ PFs < 220 km)	**Extremely Large** (220 km ≤ PFs)	**Extreme rain** Rconv > 30 mm/hr
TRMM RPFs							
R_MINOR	6.59E + 06	7.58E + 05 (***11.5%*** ^a^) 55.6%^a^	4.37E + 05 (57.7%) 26.7%	2.91E + 05 (38.4%) 48.4%	2.69E + 04 (3.5%) 20.8%	2.74E + 03 (0.4%) 4.1%	1.92E + 02 (0.025%) 0.126%
R_MEAN	***6.59E + 06***	9.62E + 05 (***14.6%*** ^a^) 57.3%^a^	5.96E + 05 (61.9%) 30.3%	3.37E + 05 (35.0%) 47.8%	2.60E + 04 (2.7%) 17.3%	3.37E + 03 (0.4%) 4.6%	2.11E + 02 (0.022%) 0.027%
R_MAJOR	6.59E + 06	2.12E + 06 (***32.2%*** ^a^) 82.4%^a^	9.92E + 05 (46.7%) 15.4%	1.02E + 06 (47.9%) 47.1%	8.81E + 04 (4.1%) 19.5%	2.65E + 04 (1.3%) 18.0%	3.20E + 02 (0.015%) 0.095%
GMMF RPFs							
NX256_1KM	***1.06E + 07***	3.75E + 06 (35.3%^a^) 83.2%^a^	1.82E + 06 (48.7%) 22.1%	1.75E + 06 (46.7%) 61.0%	1.23E + 05 (3.3%) 10.8%	4.83E + 04 (1.3%) 6.1%	1.39E + 04 ***(0.37%) 0.51%***
NX128_2KM	**8.96E + 06**	4.30E + 06 (48.0%^a^) 89.6%^a^	2.24E + 06 (52.2%) 23.9%	1.89E + 06 (44.0%) 61.3%	1.22E + 05 (2.8%) 9.7%	4.38E + 04 (1.0%) 5.1%	1.89E + 04 ***(0.44%) 0.68%***
NX64_4KM	***6.97 + E06***	4.54E + 06 (65.1%^a^) 96.0%^a^	2.22E + 06 (49.0%) 18.1%	2.13E + 06 (46.8%) 65.9%	1.41E + 05 (3.1%) 10.6%	4.86E + 04 (1.1%) 5.4%	2.15E + 04 ***(0.47%) 0.84%***

*Note*. All percentages in the table are with respect to the total number of PFs ≥ 12 km except for the percentages in the third column, which are with respect to all PFs, including small features   <   12 km. In each table cell, the value of the first, second, and third row are number, number percentage, and 2D rainfall contribution (%), respectively. The number and percentage of PFs with a mean convective rain rate (Rconv)  >  30 mm/hr are shown in the last column. Values mentioned in the main text are highlighted in bold‐italic font with their corresponding category shown in color.

^a^ Percentage with respect to all PFs including PFs   <   12 km that are ignored in this study.

**Figure 3:**
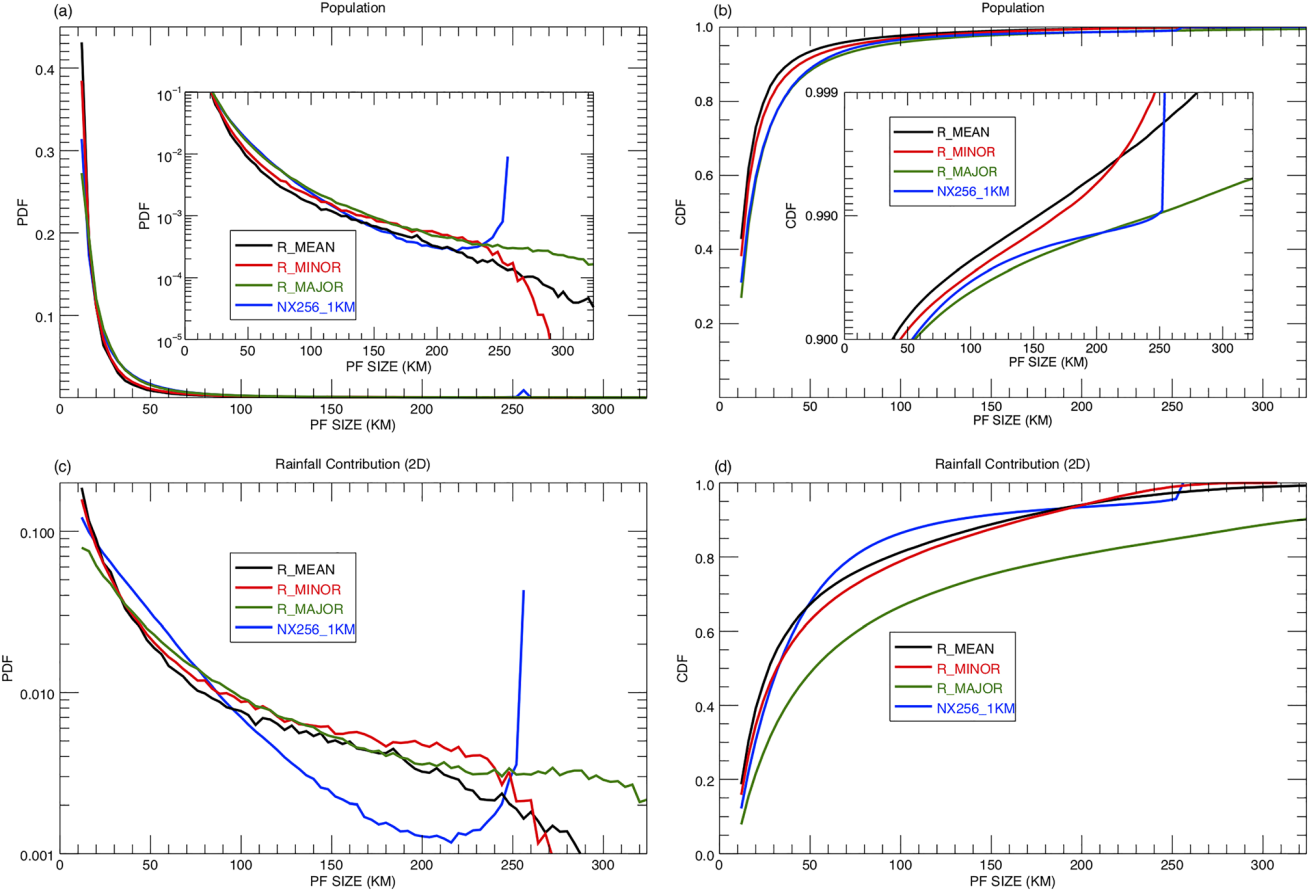
Annual (left) normalized probability density functions (PDFs) and (right) cumulative distribution functions (CDFs) of PFs with a size ≥ 12 km as a function of system size from the TRMM RPFs product and the GMMF control simulation (NX256_1KM) over the Tropics and Subtropics (36°S–36°N): (first row) population, (second row) rainfall contribution based on the 2D framework. TRMM observed RPFs are sorted with respect to three different sizes (R_MEAN, R_MINOR, and R_MAJOR) with a bin size of 4 km. To improve readability, Figures 3a and 3b also include a logarithmic *y*‐axis plot for the bottom and top 10 percentiles, respectively.

Figure 3a shows the probability density function (PDF) of PFs aggregated over the Tropics and Subtropics (36°S–36°N) for a 1‐year period (2007) as a function of rain system size from the TRMM observations and the GMMF control experiment (NX254_1KM). The TRMM RPFs are classified by their three different length scales (R_MAJOR, R_MINOR, and R_MEAN) with a bin size of 4 km. In general, the three observed distributions agree very well with the GMMF simulation; all have peak values at the smallest size (12 km) and decrease exponentially toward large‐sized PFs. The R_MEAN and R_MINOR distributions have greater population fractions (0.43 and 0.38) than R_MAJOR (0.27) and the model (0.31) at the smallest size. Due to the limited domain size (256 km) and cyclic boundaries in the control run, there is an apparent distortion of PFs ≥ 220 km as the PDF notably increases toward larger PFs and the cutoff at 256 km. The largest features with a 256‐km size account for 0.91% of the total population, much larger than the observations. Figure 3b shows the cumulative distribution functions (CDFs) of the PFs as a function of size. The simulated CDF is very close to the R_MAJOR but less than the R_MEAN and R_MINOR for all feature sizes. For R_MAJOR, about 0.91% of the total population is for PFs ≥ 256 km.

PDFs and CDFs of 2D volumetric rainfall discussed in section 2.3 are shown in Figures 3c and 3d. The observed and simulated PDFs decrease exponentially toward large‐sized PFs with small features having more volumetric rainfall than large features due to their population. The simulated rainfall contribution is less than the R_MEAN and R_MINOR for small features (  <  20 km) due to lower population numbers (Figure 3a). There is again an apparent distortion for simulated PFs ≥ 220 km as the PDF increases with size and is cutoff at 256 km. Although simulated features with a size of 256 km only account for 0.91% in population, they contribute about 4.5% of the total rainfall due to their size. Although the population curves of the simulated PFs are similar to the R_MAJOR (Figures 3a and 3b), likely for different reasons, the CDFs of rainfall contribution (Figure 3d) show the R_MAJOR distribution is very different from the R_MEAN and R_MINOR as well as the control simulation, indicating the importance of choosing a length scale for PFs.

Using the differences in the PDFs of normalized (value at each bin is divided by the total value) rainfall contribution (Figure 3c) between R_MEAN (black line) and the control experiment (blue line), PFs can be classified into approximately four size ranges: small (12 ≤ PFs   <   20 km) where the simulation has less population and rainfall contribution, medium to large (20 ≤ PFs   <   90 km) where the relative rainfall is overestimated, very large (90 ≤ PFs   <   220 km) where the rainfall contribution is underestimated, and extremely large (220 km ≤ PFs) where the PDFs of population and rainfall contribution increase as PF size increases, indicating a distortion due to the model's limited cyclic domain. Although this categorization is mainly based on the model bias in the PDF of rainfall contribution, the vertical structure and rain characteristics of PFs also support this classification and are discussed in section 3.3. The boundaries of these four categories depend on the CRM domain size and are discussed in section 4.2. The values used here are specific to simulations with a domain size of 256 km (i.e., NX256_1KM, NX128_2KM, and NX64_4KM). The observed and simulated population and 2D rainfall contribution (%) for these four size ranges are summarized in Table 2.

**Figure 4:**
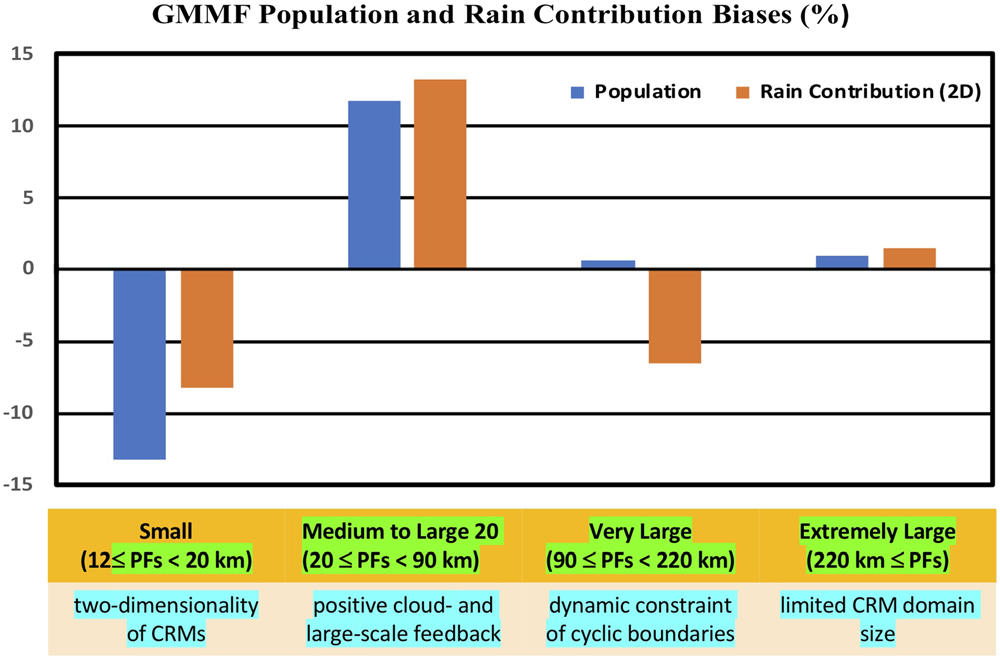
Annual normalized population and rainfall contribution differences (in %) between the GMMF control simulation and the TRMM R_MEAN product for four size categories and four different possible mechanisms that account for the model biases in each category.

Figure 4 depicts the model biases in population and 2D rainfall contribution using the R_MEAN as a reference. For small features, the simulation is about 13.2% (8.2%) less in population (rainfall contribution) than the R_MEAN. Most of the biases in the small PFs are compensated for in the medium to large PFs where the simulation is about 11.7% (13.2%) higher in population (rainfall contribution). For very large features, the model has a slightly larger population (0.6%) but less rainfall (6.5%), and for extremely large PFs, the simulation is about 0.9% (1.5%) higher in population (rainfall contribution). Four possible mechanisms that might account for these model biases are also listed in Figure 4 and will be discussed in sections 3.4 and 3.5.

### 3.2 Population and Rainfall Contributions of PFs in Selected Regions With Large Rain Biases

**Figure 5:**
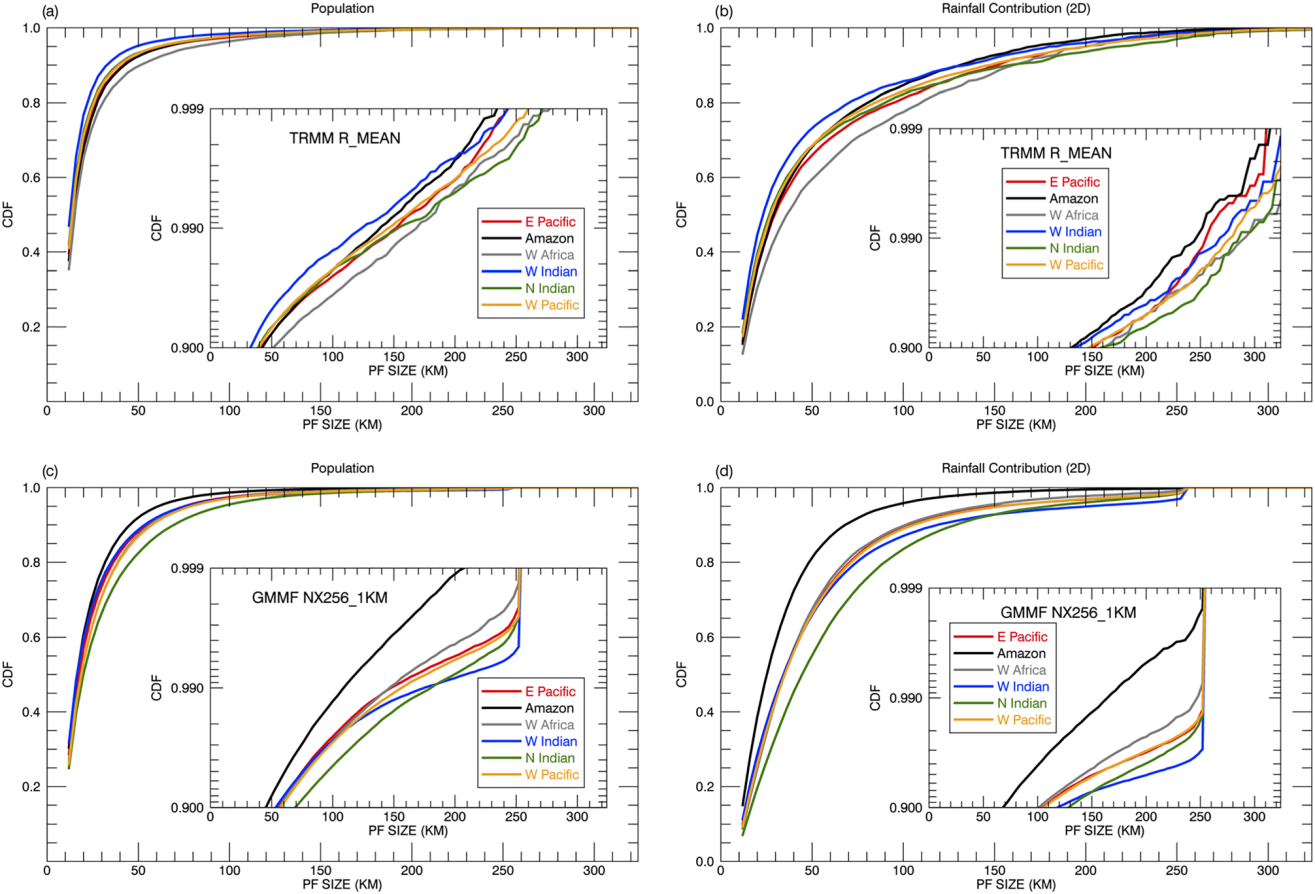
Annual normalized CDFs of (left) population and (right) regional 2D rainfall contribution of PFs ≥ 12 km as a function of size from the TRMM R_MEAN product and the GMMF control simulation (NX256_1KM) for four selected regions with positive rain biases over ocean (i.e., the tropical eastern Pacific Ocean [box A], the western Indian Ocean [box D], the northern Indian Ocean [box E], and the tropical western Pacific Ocean [box F]) and two regions with negative rain biases over land (i.e., the Amazon [box B] and western Africa [box C]). A map of the six selected regions is depicted in Figure 2c. A logarithmic *y*‐axis plot for the top 10 percentiles is embedded in each figure to improve readability.

Figure 5 shows normalized CDFs of population and 2D rainfall contribution as a function of PF size from four selected regions (shown in Figure 2c) with positive rain biases over ocean, the tropical eastern Pacific Ocean (box A), the western Indian Ocean (box D), the northern Indian Ocean (box E), and the tropical western Pacific Ocean (box F), and two regions with negative rain biases over land, the Amazon (box B) and western Africa (box C). The model tends to underestimate (overestimate) the population and rainfall contribution for small (medium to large) PFs in these regions. TRMM has more occurrences and greater rainfall contributions from large (small) PFs over western Africa (the western Indian Ocean), whereas the model has more occurrences and greater rainfall contributions from large (small) PFs over the northern Indian Ocean (the Amazon). Simulated PFs are smaller than the observed over the Amazon and western Africa due to systematic dry biases in the model. The effect of a limited domain on the population and rainfall contribution of PFs is quite small in the Amazon region because few simulated PFs develop to that size.

### 3.3 Geographical Distributions and Rain Characteristics of PFs

**Figure 6:**
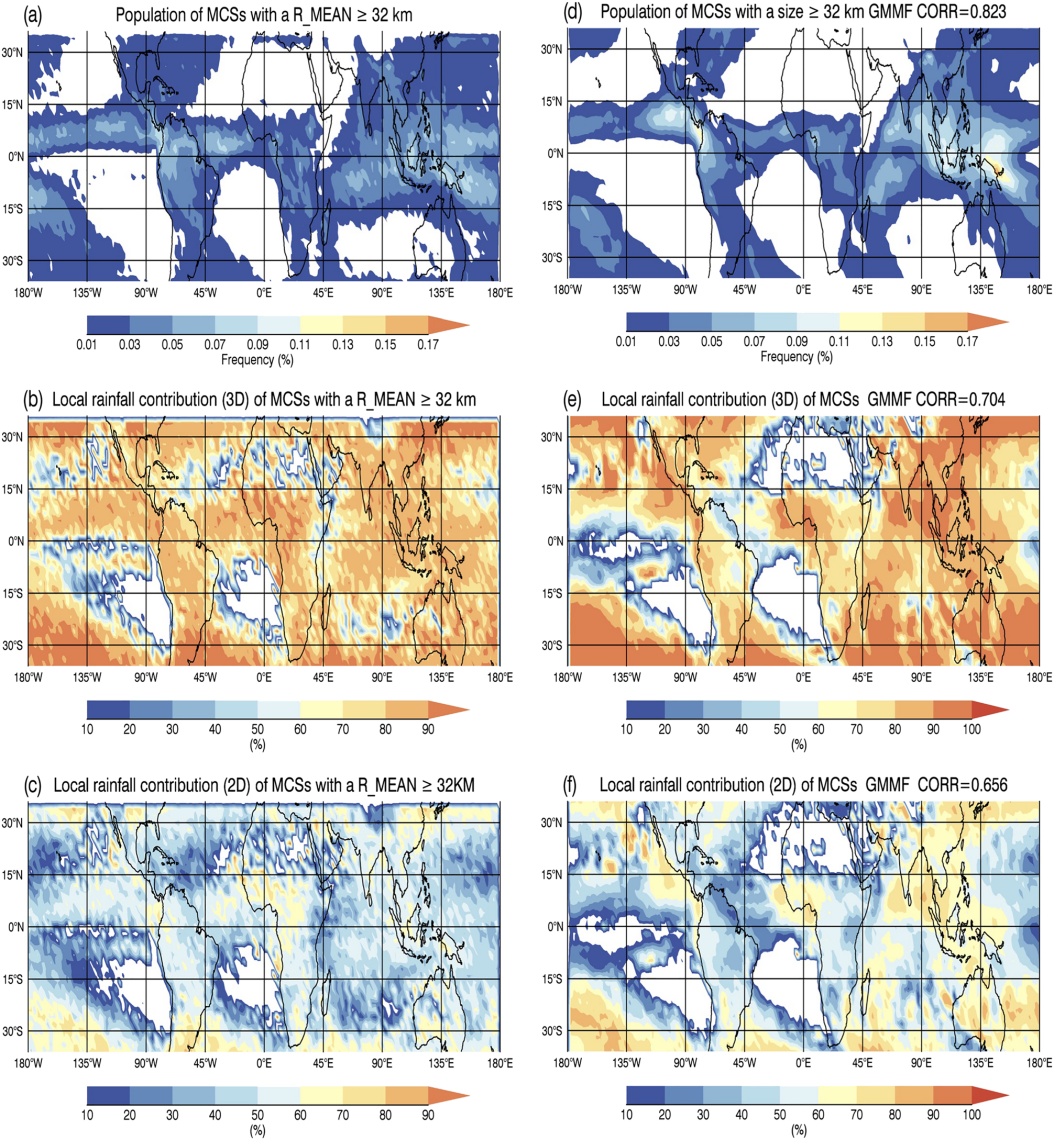
(a) Population distribution of PFs with a mean size (R_MEAN) ≥ 32 km (~1,000 km^2^ in area) with at least 1 convective pixel. The Level‐2 TRMM RPFs data are mapped to the GEOS 2.0°  ×  2.5° (lat‐lon) grid boxes; relative percentages sum to 100%. TRMM orbital sampling biases have been removed by using the reciprocal of the total number of observed pixels in each box as weighting. The local rainfall contribution of PFs with R_MEAN ≥ 32 km with at least 1 convective pixel at each GEOS grid box in the (b) 3D and (c) 2D frameworks. (d–f) Same as (a–c) except from the GMMF control experiment (NX256_1KM).

Convective precipitation features with a contiguous rain area greater than 1,000 km^2^ (~32 km in length) have been frequently classified as mesoscale convective systems (MCSs) in the literature (Cifelli et al., 2007; Houze, 1993, 2004; Liu et al., 2008; Thatcher et al., 2012). Figures 6a and 6d show the geographical distribution of large convective PFs with a size ≥ 32 km for both R_MEAN and the control run with percentages adding to 100% in each figure. The Level‐2 TRMM and simulated RPF data have been mapped to the GEOS model grids at 2°  ×  2.5° (lat‐lon) resolution. TRMM PR orbital sampling biases that have more measurements at midlatitudes have been removed in Figure 6a by using the reciprocal of the total number of observed pixels in each box as a weighting factor. Both the observed and simulated patterns are in good agreement, spatial correlation coefficient (CORR  =  0.823), with MCSs located in environments favorable for organized precipitation such as the Intertropical Convergence Zone (ITCZ), the South Pacific and South Atlantic convergence zones, the Maritime Continent, within the storm tracks of extratropical cyclones, downstream of the continents, western Africa, and the Congo and Amazon basins. The rainfall contribution from MCSs (Figures 6b and 6e) can reach 60–90% of the local total rainfall in the 3D framework. Note that in the 3D framework, each simulated PF is assumed to be circular in shape, and its volumetric rainfall equals to the product of its circular area and mean rain rate. Despite this ad hoc shape assumption, the spatial correlation coefficient reaches 0.704. This diagnostic is only used here to demonstrate that the local rainfall contribution pattern and amount of MCSs is in good agreement with observational studies in the literature (Liu et al., 2008; Nesbitt et al., 2006). The 2D rainfall contribution diagnostic, which does not have this ad hoc assumption, will be used in the rest of this paper. Figures 6c and 6f show the 2D local rainfall contribution of MCSs is considerably reduced. In this 2D framework, the volumetric rainfall of each PF is proportional to its length instead of its area; as a result, large features contribute less rainfall.

**Figure 7:**
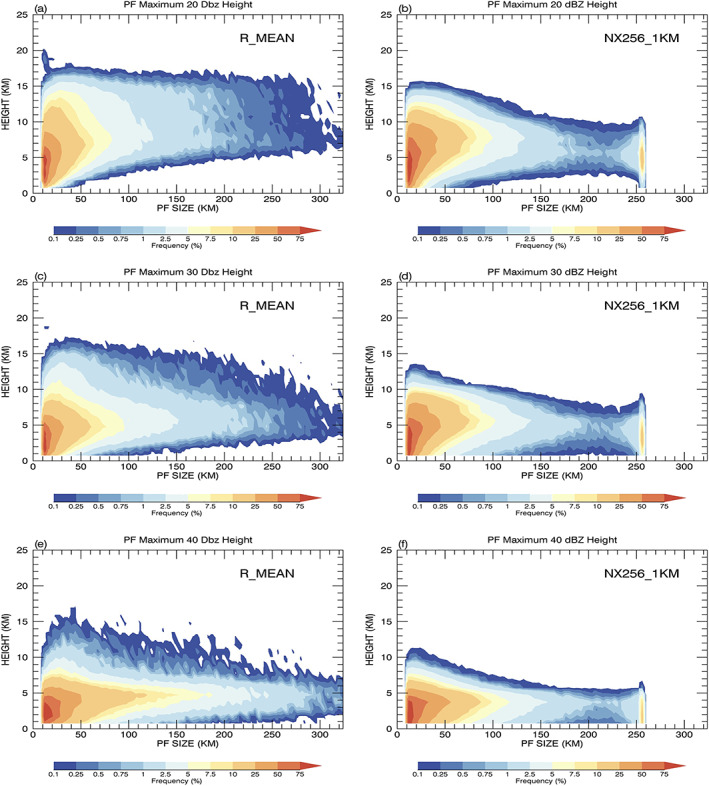
Cumulative 2D histogram of maximum (first row) 20, (second row) 30, (third row) 40 dBZ echo top heights as a function of PF size, from (left) TRMM R_MEAN observations and (right) the GMMF control simulation (NX256_4KM).

Figure 7 shows cumulative 2D histograms of maximum 20, 30, and 40 dBZ echo top heights as a function of PF size. The model rarely produces PFs with a maximum 20 dBZ echo top higher than 15 km (Figure 7b), which are frequently observed by the TRMM PR (Figure 7a). The discrepancy of having simulated radar echoes that are too weak near storm top for intense convective systems is consistent with many previous stand‐alone CRM simulations using observed large‐scale forcing from various field campaigns (Lang et al., 2011, 2014; Li et al., 2018). Lang et al. (2011) suggested the ice particle size distribution in the bulk microphysics cannot represent entrainment effects near observed storm top wherein dry air disproportionately sublimates small particles while preserving relatively large particles. The observed maximum echo top height distribution remains almost the same (~17 km) regardless the system size while the simulated one tends to lower with PF size (Figures 7a and 7b). This indicates that the intensity of simulated convection is reduced toward larger PFs. Alternatively, contributions from the stratiform precipitation columns to the maximum echo top heights could be underestimated, because the empirical size distribution for snow aggregates in the Goddard 4ICE scheme appears to be too small at colder temperatures in comparison with aircraft observations and polarimetric radars (Matsui et al., 2019). The GMMF‐simulated distributions of maximum 30 and 40 dBZ echo top heights (Figures 7d and 7f) decrease toward larger PFs in reasonable agreement with the TRMM observations, but the observations still have more PFs with a maximum echo top higher than in the simulation. The conventional CRM domain size (256 km) of MMFs is too small and cannot cover all of the observed PFs. There is an apparent cutoff at a size of 256 km and some distortion of the relative occurrence frequency of PFs with a size (from 220 to 256 km) near the domain size limit.

**Figure 8:**
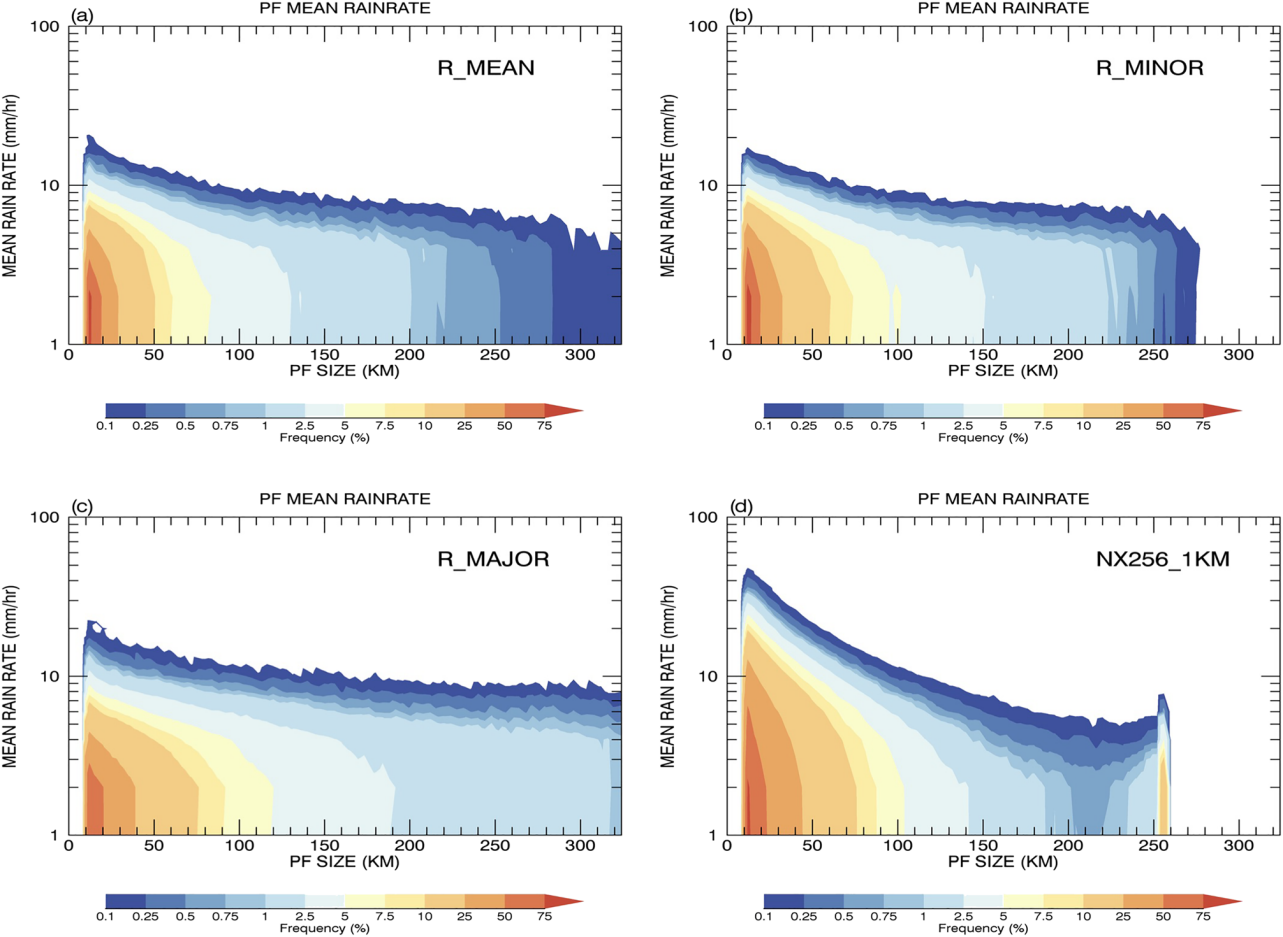
Cumulative 2D histogram of mean PF rainfall rate from the TRMM RPFs product (a–c) as a function of three different PF sizes (R_MEAN, R_MINOR, and R_MAJOR) and (d) the GMMF control simulation (NX256_1KM).

Figure 8 shows cumulative 2D histograms of mean PF rainfall rates as a function of system size. Observations rarely produce PFs with a mean rainfall rate greater than 20 mm/hr; also, the storm morphology does not affect the magnitude of rain intensity only the size distribution (Figures 8a–8c). The R_MAJOR histogram extends to large sizes whereas the R_MINOR rarely has PFs  >  256 km. Figure 8d shows that the simulation overestimates the occurrence frequency of heavy precipitation ( > 20 mm/hr) for the small PFs, whereas it underestimates the rain rate for the very large PFs.

### 3.4 Effects of a Limited Domain and Cyclic Lateral Boundaries

The extremely large category (PFs ≥ 220 km) is defined as PFs whose sizes approach that of the model domain. The normalized PDFs of population in this category increase as PF size increases, while the observed PDFs decrease exponentially as PF size increases. Apparent distortions are also found in the vertical structure and mean precipitation rate of these PFs (Figures 7 and 8) due to the effect of a limited model domain. Two possible mechanisms might explain this positive bias in population. First, MCSs and extratropical frontal systems usually have horizontal scales of a few hundred kilometers, which exceeds the conventional CRM domain size of 128 or 256 km in MMFs. In the CRMs, these systems will be represented and counted in the population of PFs whose size is equal to the domain size. Second, the periodicity of the CRM domain prevents PFs from interacting directly with the environment in neighboring GCM grid boxes. The life span of extremely large PFs will be prolonged due to the lack of a drier air mass being entrained into these systems. Despite the mean precipitation intensity of extremely large PFs being less than the observed (Figure 8), their normalized rainfall contribution is overpredicted (Figures 3 and 4) mainly due to their large population.

**Figure 9:**
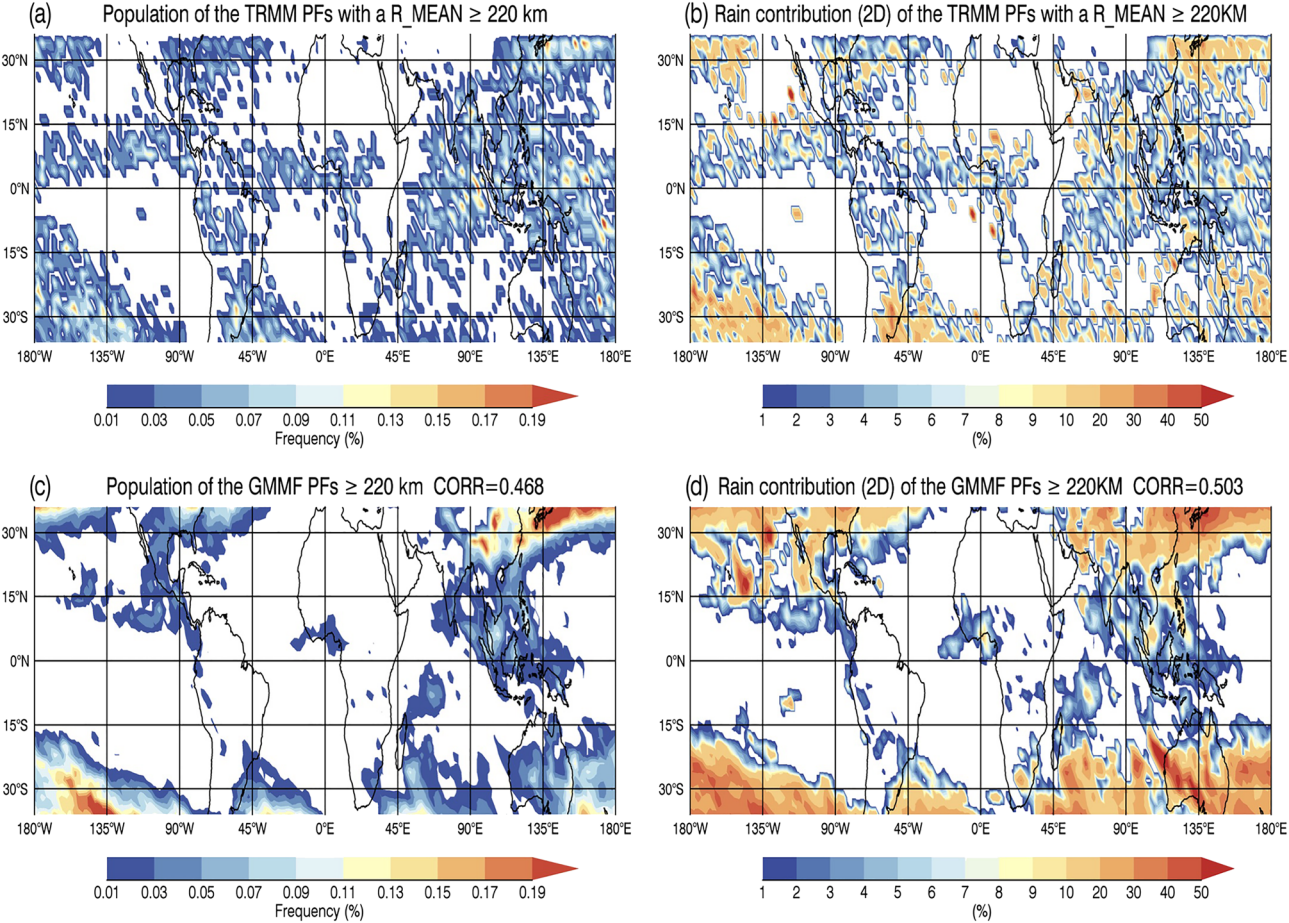
(a) Population distribution of PFs with a mean size (R_MEAN) ≥ 220 km. The Level‐2 TRMM RPF data are mapped to the GEOS 2.0°  ×  2.5° (lat‐lon) grid boxes; relative percentages sum to 100%. TRMM orbital sampling biases have been removed by using the reciprocal of the total number of observed pixels in each box as weighting. The local rainfall contribution of PFs with R_MEAN ≥ 220 km at each GEOS grid box in the 2D framework. (c–d) Same as (a, b) except from the GMMF control experiment (NX256_1KM).

Figure 9 shows the observed and simulated geographical distributions and local rain contributions from these extremely large PFs. Most of the observed and simulated extremely large PFs occur in the Subtropics. However, the model overestimates their normalized occurrence frequency and local rainfall contribution especially over the western Pacific storm track region and the South Pacific Convergence Zone (SPCZ). The TRMM observations have more extremely large PFs within the ITCZs that account for up to 10% of the local rainfall. In the model, extremely large PFs in the Tropics are mainly located over the Maritime Continent and the Indian Ocean with a local rainfall contribution of up to 10%. The simulated normalized population and rainfall contribution are considerably underpredicted within the ITCZs. The observed and simulated geographical distributions of PFs for the other size categories (i.e., small, medium to large, and very large) and two examples of PFs with a domain‐wide size are provided and described in Supporting Information S1.

Very large PFs (~90–220 km) are defined as large PFs whose simulated rainfall contribution is underestimated and whose population decreases as size increases (Figures 3 and 4). The observations have more intense convection (Figure 7) and larger precipitation rates (Figure 8) than the control simulation within this size range. Geographically, the simulated occurrence of very large PFs (Figures S1e and S1f) and extremely large PFs (Figures 9a and 9c) are significantly underpredicted in the ITCZs. One possible mechanism to account for this model bias is the dynamic constraint imposed in the CRM with a cyclic boundary condition. The periodicity of the CRM domain implies that there is no domain average mass convergence or divergence. Hence, the simulated domain mean upward mass flux must be balanced by compensating subsidence within the same bounded domain. For most stand‐alone CRM simulations, the domain size has been predetermined to be sufficiently larger than the size of the target precipitation system such that the domain mean mass flux is zero and the compensating subsidence develops outside the target cloud system. However, an MMF needs to have the ability to simultaneously represent precipitation systems of all sizes at different geographical locations. The dynamic constraint will force downdrafts to coexist with the updrafts within very large and extremely large PFs and reduce their convective intensity, size, and precipitation rate. As a result, the model cannot support very large and extremely large intense convective systems and underestimate their population in the ITCZs. An example of a PF with a 256‐km domain size is presented in Figures S3c and S3d to demonstrate this artificial dynamic constraint.

### 3.5 Population Bias of Small PFs and Excessive Rain Bias of Medium to Large PFs

One of the apparent deficits in the control simulation is the underestimation of the relative occurrence of small PFs (12 ≤ PFs   <   20 km). Li et al. (2018) studied the evolution of precipitation structure for an MJO event during the DYNAmics of the MJO (DYNAMO) field campaign using ground based, ship‐borne, and spaceborne precipitation radars and a 3D CRM. The 30‐day simulation from a 3D GCE with 256  ×  256  ×  30 grid points and 1‐km horizontal grid spacing realistically reproduced the observed PDF of PFs from the TRMM PR. The simulation slightly overestimated PFs less than 400 km^2^ (~20 km) by less than 1% (Figure 9 of Li et al., 2018). This study suggests that the significant underprediction (~13.2%) of the small PF population in the GMMF (Figures 3 and 4) might come from the use of 2D GCEs. Although the observed and simulated PDF curves in Figure 3a are normalized, the observations have one more degree of freedom to support more concurrent small PFs than the 2D simulations. The two‐dimensionality has more impact on the frequency of small PFs than that of large PFs. Note the two‐dimensionality mechanism in this study primarily effects the PF count. It also plays a key role in the turbulence (Kahn et al., 2011; Moeng et al., 2004), vertical velocity (Redelsperger et al., 2000; Zeng et al., 2007), entrainment (Petch et al., 2008), and microphysics and radiation (Phillips & Donner, 2006). These too will likely affect the simulated PFs but are beyond the scope of this study.

**Figure 10:**
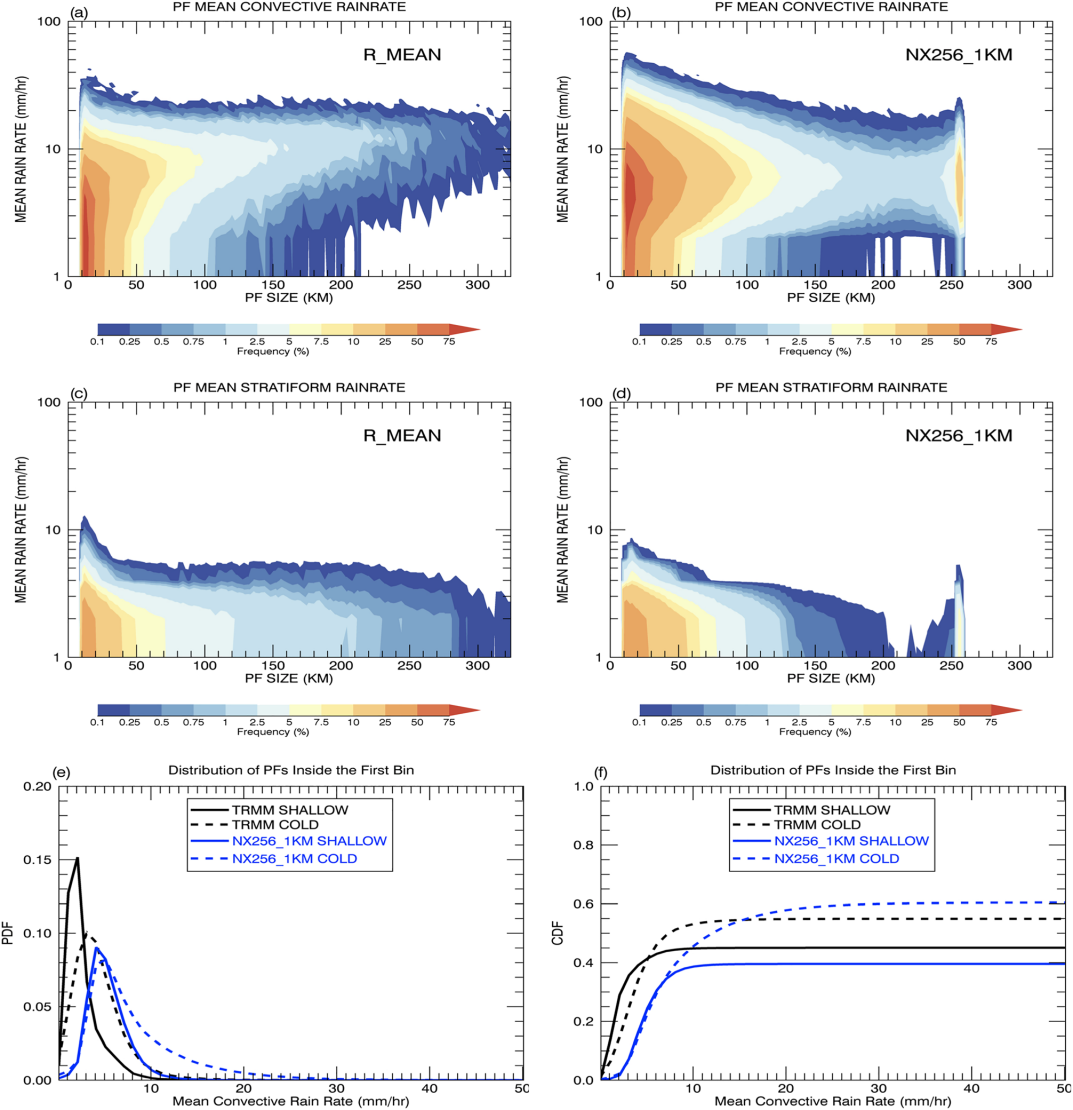
Cumulative 2D histograms of mean rainfall rate for (a) convective and (c) stratiform pixels as a function of PF size from TRMM R_MEAN observations and (b) and (d) the GMMF control simulation. Normalized (e) PDFs and (f) CDFs of observed and simulated shallow (cold) convection defined by having a PF maximum echo top height less (greater) than 4.5 km for the first size bin.

The mean precipitation rate for the small and medium to large categories is overestimated (Figure 8). The observed and simulated rainfalls are further categorized into stratiform and convective rain using the algorithms discussed in section 2.2. Cumulative 2D histograms of mean convective (stratiform) rain rate from convective (stratiform) pixels inside each PF are shown in Figures 10a–10d. The extreme mean rain rates in the simulation shown in Figure 8 mainly come from convective rain that exceeds 30 mm/hr for PFs   <   90 km. Also, model PFs with mean rain rates less than 3 mm/hr tend to be classified as stratiform instead of convective systems. For the very large features (PFs  >  90 km), the observed mean convective rain increases as the size increases, whereas the simulated rain remains about the same and is underestimated. The simulated stratiform rain rates for small PFs are less than the observed. This might be due to observed uncertainties and the difference in the convective‐stratiform separation algorithms as discussed in section 2.2. The occurrence of stratiform rain is also underestimated for the very large PFs. To investigate further the extremely heavy and light convective precipitation, the smallest PFs are separated into shallow and cold convection with maximum echo top heights below and above 4.5 km, respectively. Cold convection, including mid‐level and deep, is under the influence of ice microphysics, whereas the shallow convection is dominated by warm rain processes. Figures 10e and 10f show PDFs and CDFs of the observed and simulated shallow and cold convection. The observed mean PF convective rain rates have strong peaks at 2 and 3 mm/hr, while the model is skewed toward larger values with peaks at 4 and 5 mm/hr for shallow and cold convection, respectively. The simulation produces much fewer occurrences of light rain less than 2 mm/hr, and the simulated cold convection has a long tail toward heavy rain rates of 30 mm/hr. Figure 10f shows observed shallow convection accounts for ~45% of the rain for the smallest PFs while for the simulation it is ~40%.

**Figure 11:**
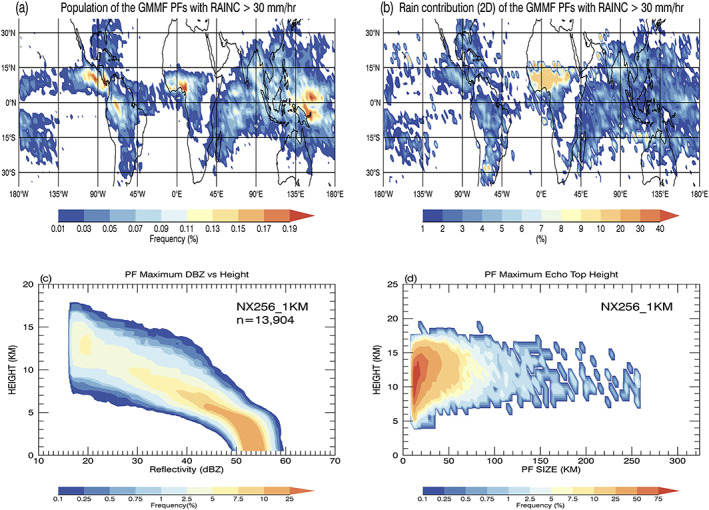
(a) Population distribution of PFs with extreme mean convective rain rates ≥ 30 mm/hr from the GMMF control simulation. The relative percentages in the panel sum to 100%. (b) Local rainfall contribution at each GEOS grid box from PFs with extreme convective rain using the 2D framework. (c) Joint histograms of maximum radar reflectivity and height with the frequency respect to the total number of PFs with extreme convective rain (*n*  =  13,904). (d) Cumulative 2D histogram of maximum echo top heights as a function of PF size.

Table 2 shows that the observed occurrence and rainfall contribution of PFs with extreme convective rain are at least one order smaller than in the control simulation over the whole Tropics and Subtropics. The simulated PFs still only contribute to 0.37% (0.51%) of the total population (rainfall) over the Tropics and Subtropics. Figures 11a and 11b show the geographical distribution of simulated extreme convection and their local rainfall contribution. Most of the population is located over the Tropics and accounts for less than 10% of the local rain. PFs with extreme rainfall are mainly small to large cold convection with a maximum echo top height greater than 4.5 km and a size less than 90 km (Figures 11c and 11d). The ice microphysical processes are important for cold convection but might not be the cause of the extreme rainfall; Chern et al. (2016) showed that using different ice microphysical schemes in the GMMF only changed the amount of excessive rainfall biases slightly. In MMFs, the embedded CRMs are driven by the simulated large‐scale forcing from a “free‐running” GCM, and feedbacks between the large‐scale circulation and cloud‐scale processes can fully interact. Tao and Chern (2017) showed that GMMF simulations had a more intense Hadley circulation due to a positive large‐scale surface evaporation and wind feedback that transported the moisture from the Subtropics to the tropical ITCZs and SPCZ where the heavy rainfall occurred. Also, rain drop sizes could be overestimated with excessive rain mass in the 4ICE scheme due to the Marshall‐Palmer size distribution. Currently, the 4ICE scheme does not have an upper limit to the slope parameter for the exponential size distribution, which can cause unrealistically large raindrops and consequently large rainfall rates in convective cells as was evaluated from the polarimetric radar measurements (Matsui et al., 2019).

## 4 GMMF Sensitivity Experiment Results

### 4.1 Effect of CRM Horizontal Grid Spacing

**Figure 12:**
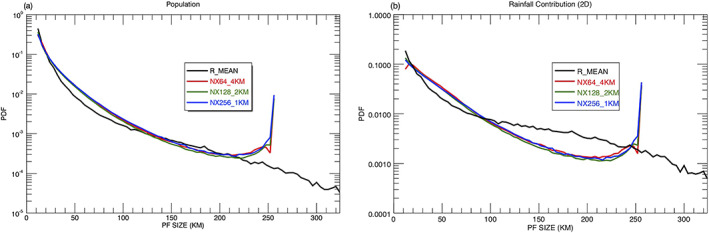
Normalized PDFs of (a) population and (d) rainfall contribution (based on the 2D framework) of observed and simulated PFs for three GMMF sensitivity experiments as a function of size over the Tropics and Subtropics (36°S–36°N). The three GMMF experiments (NX64_4KM, NX128_2KM, and NX256_1KM) have the same CRM domain size (256 km) but different grid resolutions of 4, 2, and 1 km. To compare with the TRMM PFs with a pixel size of ~4.3 km, the GMMF output of the high‐resolution runs (i.e., NX128_2KM and NX256_1KM) are averaged to 4‐km grid boxes.

Three GMMF experiments with the same CRM domain size (256 km) but different GCE horizontal grid spacing (i.e., 1, 2, and 4 km) are performed (Table 1). To compare with TRMM observations and other GMMF runs with 4‐km grid spacing, output from the high‐resolution runs (i.e., NX256_1km and NX128_2km) is averaged to 4‐km grid boxes. As a result, details of small PFs   <   4 km are lost. Experiments with higher resolution CRMs tend to shift PF population distributions toward smaller sizes. Table 2 shows the total population of PFs increases as the grid spacing decreases, but there is a net decrease when small PFs   <   12 km are ignored. Figure 12 shows the normalized PDFs, and rainfall contributions are very similar among the three experiments. The four PF size categories and their boundaries do not change with model grid spacing. Their population and rainfall contribution only change slightly as shown in Table 2. The distribution of rain rates and maximum radar echo top heights are also quite similar (not shown). The population and rainfall contribution of PFs with extreme convective rain rates ( > 30 mm/hr) does increase slightly as the grid spacing increases (Table 2).

### 4.2 Effect of CRM Domain Size

**Figure 13:**
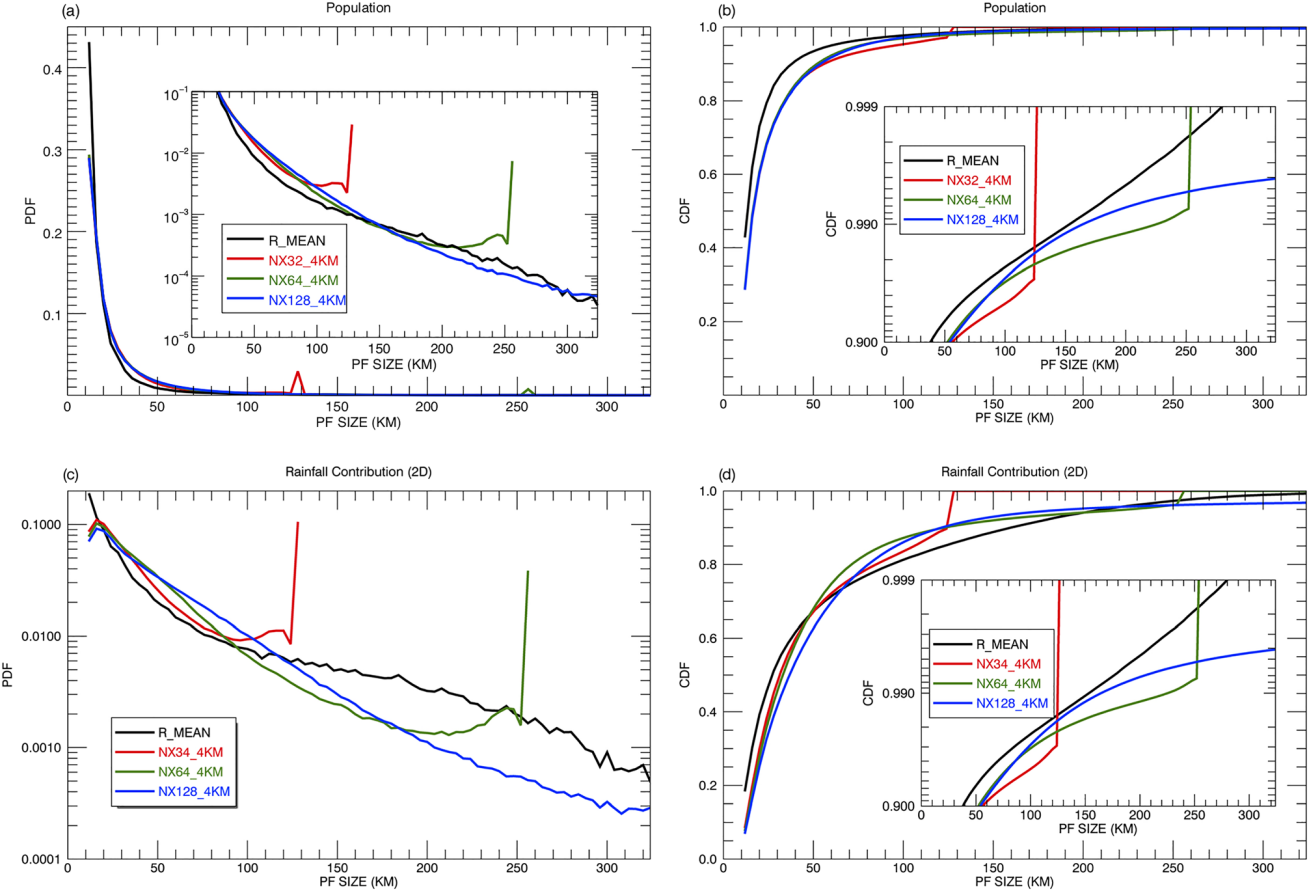
Annual (left) normalized PDFs and (right) CDFs of the populations and rainfall contribution (based on the 2D framework) of observed TRMM PFs in terms of mean size (R_MEAN) and simulated PFs for three GMMF sensitivity experiments (NX32_4KM, NX64_4KM, and NX128_4KM) having CRM domain sizes of 128, 256, and 512 km as a function of size over the Tropics and Subtropics (36°S–36°N). A logarithmic *y*‐axis plot for the bottom (top) 10 percentiles is embedded in Figure 13a (Figures 13b and 13d) to improve readability.

To understand the effects of CRM domain size on the rain characteristics of PFs, three GMMF experiments (Table 1) are performed with the same model grid spacing (4 km) but different domain sizes (i.e., 128, 256, and 512 km). Table 3 shows the number of PFs increases about 70% when the domain size is doubled (second column), but the relative percentages of PFs  >  12 km remain about the same (~65%, third column). The rainfall contribution decreases as the domain size increases for small PFs (  <  20 km) while it increases as the domain size increases for PFs with extreme rain. Figures 13a and 13b show the PDFs, and CDFs of PFs are very similar except near the domain cutoffs. About 0.9% of the PFs have a size larger than 300 km in the simulation with 512‐km domains. The smaller the domain, the greater the limited domain effects become. The population (rainfall contribution) at the domain cutoff accounts for ~3% (~10%) and ~1% (~3%) of the total PFs for domain sizes of 128 and 256 km, respectively. Since PFs with a R_MEAN  >  360 km are rarely observed in the TRMM RPFs data set, a 512‐km CRM domain size is big enough to cover all PFs. Hence, the limited domain effect is negligible for the GMMF run with 512‐km CRM domains. The size boundaries for the four PF categories mentioned in section 3 are specific to simulations with a domain size of 256 km. No dimensionless reference scale for these boundaries as a function of CRM domain size can be found. Figure 13c shows the boundaries for small PFs are the same (12 ≤ PFs   <   20 km) for all three simulations. However, the boundaries for the medium to large PFs with a positive rainfall bias and the very large PFs with a negative rainfall bias increase (the intersections of the black and blue curves) in the simulation with 512‐km domains. The rainfall contribution except for the small PFs is all positive (black and red curves) in the simulation with 128‐km domains. The four above mentioned mechanisms likely still apply for the small domain simulation, but their effects on rainfall contribution overlap each other, making it difficult to identify their boundaries.

**Table 3:** Numbers, Number Percentages, and Rainfall Contribution (%) of Precipitation Features Over the Tropics and Subtropics (36°S–36°N) From the TRMM R_MEAN Observations and the GMMF Simulations Using Three Different Model Domain Sizes

Name	**All** PFs number (PFs ≥ 4 km)	**Total** (PFs ≥ 12 km)	**Small** (12 ≤ PFs < 20 km)	**Extreme rain** Rconv > 30 mm/hr
TRMM RPFs				
R_MEAN	6.59E + 06	9.62E + 05 (14.6%^a^) 57.3%^a^	5.96E + 05 (61.9%) 30.3%	2.11E + 02 (0.022%) 0.027%
GMMF RPFs				
NX32_4KM	***4.00E + 06***	2.61E + 06 (***65.2%*** ^a^) 95.6%^a^	1.26E + 06 (48.3%) ***19.8%***	6.52E + 03 (0.25%) ***0.44%***
NX64_4KM	***6.97 + E06***	4.54E + 06 (***65.1%*** ^a^) 96.6%^a^	2.22E + 06 (49.0%) ***18.1%***	2.15E + 04 (0.47%) ***0.84%***
NX128_4KM	***1.23E + 07***	7.99E + 06 (***65.0%*** ^a^) 95.5%^a^	3.87E + 06 (48.4%) ***16.3%***	4.49E + 04 (0.56%) ***0.95%***

*Note*. All percentages in the table are with respect to the total number of PFs ≥ 12 km except for the percentages in the third column, which are with respect to all PFs, including small features   <   12 km. In each table cell, the value of the first, second, and third row are number, number percentage, and 2D rainfall contribution (%), respectively. The number and percentage of PFs with a mean convective rain rate (Rconv)  >  30 mm/hr are shown in the last column. Values mentioned in the main text are highlighted in bold‐italic font with their corresponding category shown in color.

^a^ Percentage with respect to all PFs including PFs   <   12 km that are ignored in this study.

**Figure 14:**
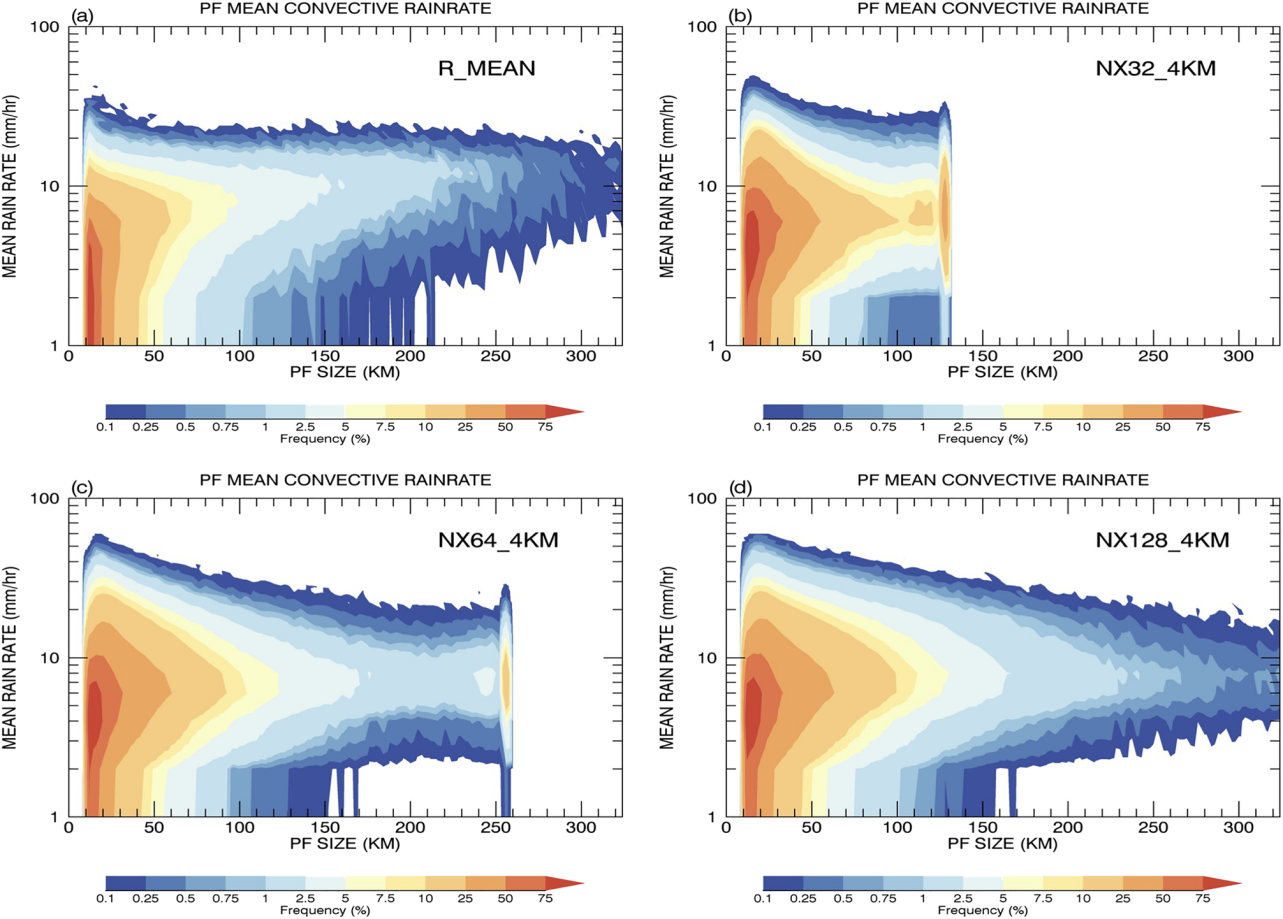
Cumulative 2D histograms of mean convective rainfall rate for convective pixels as a function of PF size from (a) the TRMM observations in terms of mean size (R_MEAN) and the GMMF simulations with three different CRM domain sizes: (b) 128, (c) 256, and (d) 512 km.

Figure 14 shows the observed and simulated cumulative 2D histograms of mean PF convective rainfall rate as a function of size. The proportion of extreme convective rain rates slightly increases as the domain size increases, and all underestimate the frequency of light convective rain less than 2 mm/hr. The GMMF with 512 CRM domains increases the rainfall distribution toward large PFs and mitigates the negative rainfall bias for the very large PFs.

## 5 Summary and Discussion

In this study, cloud and precipitation systems over the Tropics and Subtropics are simulated with the GMMF v3.0 and compared against TRMM observations. A methodology, in close analogy to the TRMM RPFs, is developed to analyze simulated cloud precipitating structures from the output of the embedded 2D GCEs. Three observed length scales (i.e., R_MINOR, R_MEAN, and R_MAJOR) are utilized to assess the uncertainty range of the definition of PF size. The distributions of population and rainfall are somewhat sensitive to the chosen length scale. They are quite similar between R_MINOR and R_MEAN but considerably different from R_MAJOR, which moves the population and rainfall contribution toward large PFs.

Despite the limitations of 2D CRMs and the use of simulated large‐scale forcing from a “free‐run” GCM, the simulated population distribution, horizontal and vertical structure of PFs, and the geographical location and local rainfall contribution of MCSs are in good agreement with TRMM observations. However, some discrepancies are found in the model simulations and can be identified and quantified within the PF distributions. Figure 4 summarizes the population and 2D rainfall contribution biases for the GMMF control simulation in Table 2. The PFs can be characterized into four categories: small, medium to large, very large, and extremely large. Four different mechanisms might account for the model biases in each category.

For small features, the two‐dimensionality of the CRMs is linked to the underestimation of population due to having one less degree of freedom to support many concurrent small PFs as observed. Despite the model's tendency to produce larger convective rain rates, the deficiency in rain contribution mainly comes from the shortfall in occurrences. Increasing the model horizontal resolution tends to produce more PFs   <   12 km, but this still cannot overcome the two‐dimensionality effect.

Most of the biases in small PFs are largely compensated for in the medium to large category. Simulated PFs in this size range play a crucial role in the overall positive tropical precipitation biases in the model due to their larger population and precipitation rate. For stand‐alone GCE simulations with “prescribed” large‐scale forcing from a field campaign, Tao and Chern (2017) showed the 2D and 3D GCEs can realistically reproduce observed precipitation. However, the large‐scale forcing applied to the embedded CRMs within an MMF derived from a “free run” host global model has inherent model biases. Because of the nonlinear interactions between the cloud‐ and large‐scale, positive feedback loops might develop. Luo and Stephens (2006) found a positive convection‐wind‐evaporation feedback loop that prevails in MMFs and fuels tropical convection by enhancing low‐level moisture convergence. Tao and Chern (2017) showed GMMF simulations had a more intense Hadley circulation due to a positive large‐scale surface evaporation and wind feedback that transported the moisture from the Subtropics to the tropical ITCZs and SPCZ. This process plays an essential role in the overestimation of precipitation in this category. The precipitation biases mainly come from convection having larger rain rates and occasional extreme ones greater than 30 mm/hr that are rarely observed in the TRMM RPFs data set.

For very large PFs, TRMM observations show the existence of strong convective cells with maximum 20 dBZ echo top heights exceeding 17 km that are not simulated by the model. The maximum frequency of observed mean convective rain increases as PF size increases, whereas the simulated rain remains about the same and is underestimated. Although the model produces a slightly higher relative population, the PFs in this size range contribute considerably less toward the total rainfall. The cyclic boundaries impose a strong artificial dynamic constraint wherein upward mass flux needs to be balanced by the compensating subsidence in a bounded domain. As a result, the model cannot support intense convective PFs in this size range, which leads to an underestimate of their occurrence in the ITCZs. PF populations tend to shift toward larger sizes for a CRM with larger domain. Increasing the CRM domain size to 512 km allows more medium to large PFs to grow bigger and slightly mitigates the rain biases, but the dynamic constraint still prevails in this size category.

The domain size of most stand‐alone CRM studies has been predetermined to be sufficiently larger than the size of the target systems to avoid distortions from lateral boundaries. The results from this study indicate that an MMF with a small CRM domain tends to produce more extremely large PFs due to distortions from lateral boundaries while reducing the occurrence of large deep convective systems in the ITCZs due to the dynamic constraint of a bounded cyclic domain. However, running an MMF with a small CRM domain is still very attractive for reducing computational expense especially for climate simulations. The acceptability of an MMF simulation all depends very much on the goals of the study, the validation matrix, and the available computing resources.

In summary, four possible mechanisms might account for the GMMF model biases: (1) the two‐dimensionality of the CRMs, (2) a positive convection‐wind‐evaporation feedback loop, (3) an artificial dynamic constraint in a bounded CRM domain with cyclic boundaries, and (4) the limited CRM domain size. The first and third mechanisms tend to reduce rainfall and are inherent constraints from the 2D CRMs with cyclic boundaries. The effect of the fourth mechanism will be diminished if a 512‐km CRM domain size is used, which is consistent with many previous stand‐alone CRM studies (Johnson et al., 2002; Petch & Gray, 2001; Tompkins, 2000). Currently, the second mechanism is the main cause of the excessive tropical precipitation biases in the GMMF. Future improvements need to focus on how to reduce the over energetic convective systems in the CRMs and the strength of the Hadley circulation in the GCM. In this study, the embedded 2D CRM domain is aligned in an east‐west direction. The CRM orientation might have an influence on simulated feature sizes. This topic is left as a future research work because substantial efforts are needed to design the necessary sensitivity experiments and conduct the GMMF simulations.

## Supporting information

Supporting Information S1

## Data Availability

The TRMM 3B43 product used in this study is accessible from the website (https://pmm.nasa.gov/data-access/downloads/trmm). The new GMMF RPFs data set and the post‐processing scripts required to reconstruct the figures and tables in this paper and the supporting information are publicly available online in the Cloud Library at the NASA Goddard data portal repository (https://portal.nccs.nasa.gov/cloudlibrary). Click “JAMES_2020_CHERN” button to download the data file.
