# Supplementary material for: Evaluating Precipitation Features and Rainfall Characteristics in a Multi‐Scale Modeling Framework
Source: J Adv Model Earth Syst. 2020 Aug 21;12(8):e2019MS002007. doi: 10.1029/2019MS002007 (PMC7507770; doi:10.1029/2019MS002007)
Supplement: Supplementary file 1 — Supporting Information S1 [file JAME-12-e2019MS002007-s001.docx]

**Supporting Information for “Evaluating Precipitation Features and Rainfall Characteristics in a Multi-scale Modeling Framework”**

**Jiun-Dar Chern^1,2^, Wei-Kuo Tao^1^, Stephen E. Lang^1,3^, Xiaowen Li^1,4^, and Toshihisa Matsui^1,2^**

^1^NASA Goddard Space Flight Center, Mesoscale Atmospheric Processes Laboratory, Greenbelt, Maryland, USA

^2^University of Maryland, Earth System Science Interdisciplinary Center, College Park, Maryland, USA

^3^Science Systems and Applications Inc., Lanham, Maryland, USA

^4^GESTAR, Morgan State University, Baltimore, Maryland, USA

**Contents of this file**

Text S1 to S2

Figures S1 to S3

**Introduction**

Tropical Rainfall Measuring Mission (TRMM) Radar-defined Precipitation Features (RPFs) product (Liu et al., 2008) and the one-year (2007) model output from the Goddard Multiscale Modeling Framework (GMMF) control simulation are used in this supporting information. Using model biases in relative population and rainfall contribution from the GMMF control run, precipitation features (PFs) can be characterized into four size categories: small (PFs < 20 km), medium to large (20 ≤ PFs < 90 km), very large (90 ≤ PFs < 220 km), and extremely large (PFs ≥ 220 km). The observed and simulated geographical distribution and local rainfall contribution from PFs in the extremely category are discussed in the main text and shown in Figure 7. Here, a corresponding analysis is provided of PFs from the other three categories in S1.Two examples of PFs with a 256 km size and the artificial dynamic constraint of a bounded cyclic domain are illustrated in S2.

**S1. Population and Rainfall Contributions in the Tropics and Subtropics for Small, Medium to Large and Very Large Precipitation Features**

. Figures S1a-d show the simulated population patterns from the GMMF control run (NX256_1KM) are in good agreement with the TRMM observations with a spatial correlation coefficient of 0.824 and 0.848 for small and medium to large PFs, respectively. The simulation slightly overestimates the relative population of medium to large PFs over the Maritime Continent and the East Pacific Intertropical Convergence Zone (ITCZ). The model underestimates the relative population of very large PFs in the Pacific and Atlantic ITCZs and overestimates them over the West Pacific and East Indian Oceans and the extratropical storm tracks with a smaller spatial correlation coefficient of 0.677 (Figures S1e-f).

Figures S2a-b show small PFs have the largest contribution to local rainfall over the subtropical subsidence regions where environments are unfavorable for organized precipitation. The simulation overestimates the rainfall contribution of medium to large PFs (Figures S2b-c) due to having large population and mean precipitation rates. In general, the simulated very large PFs with small mean rain rates contribute less to local rain than observations especially in the Pacific and Atlantic ITCZs due to an artificial dynamic constraint of using a bounded CRM domain with cyclic boundaries (Figures S2e-f).

# S2. Radar Reflectivity and Mass Fluxes of GMMF PFs with a Horizontal Size of 256 km

Figures S3a-b show simulated radar reflectivity cross sections of two 256km-wide PFs that occurred at the same time (0000 UTC 3 January 2007) but at different locations. The first PF is a low-level stratiform rain system covering the whole domain; the second is a deep convective system surrounded by stratiform rain. To illustrate the artificial dynamic constraint of using a CRM with a bounded cyclic domain, Figures S3c-d show the vertical velocity and the domain average upward and downward mass fluxes of the PF shown in Figure S3b. A CRM with a cyclic boundary condition implies there is no domain mean mass convergence or divergence. Hence, the domain average upward mass flux must be balanced by compensating subsidence as demonstrated in Figure S3d. Figure S3c shows the coexistence of upward and downward motion inside a large convection system, which reduces its intensity, convective area and mean precipitation rate.


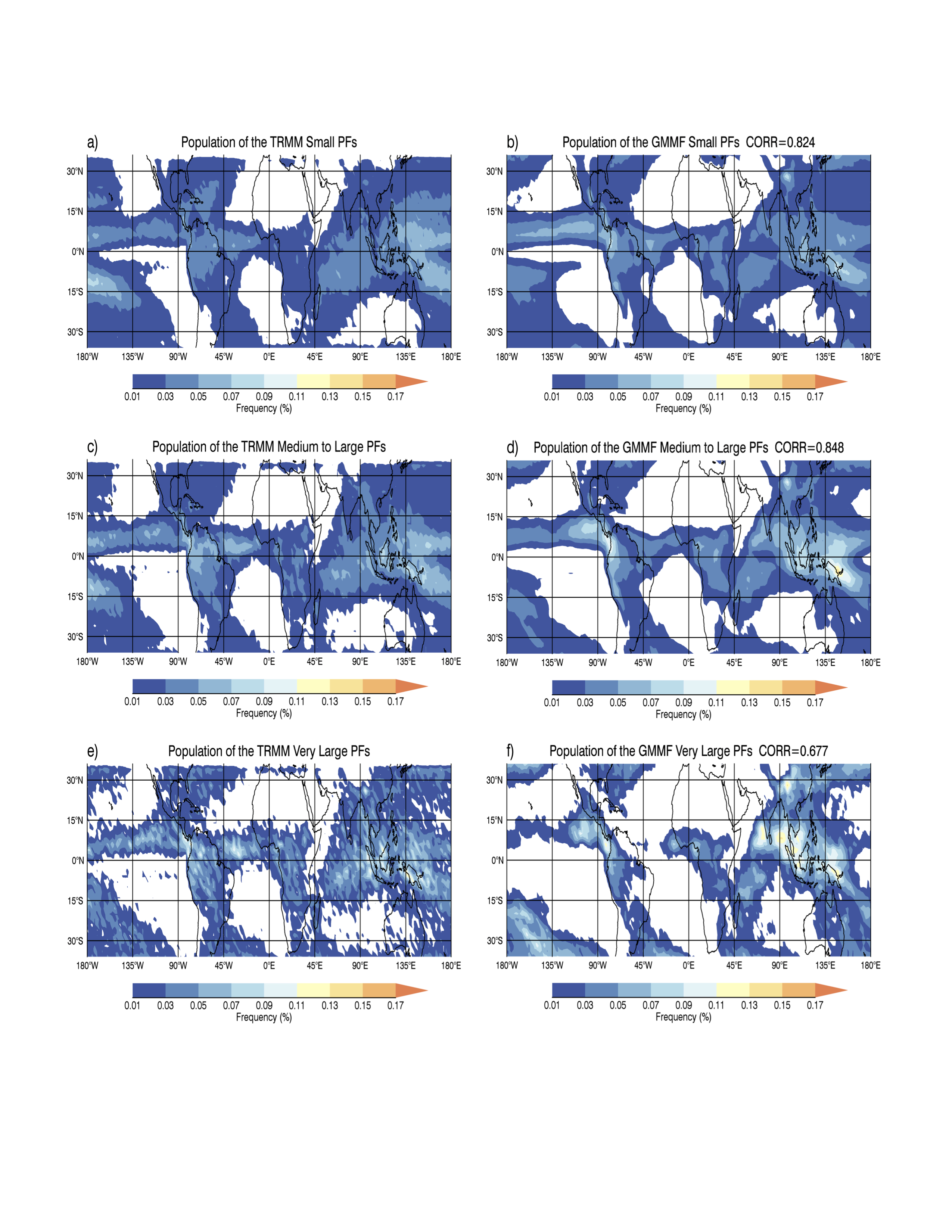


**Figure S1.** Annual normalized population distribution from the (left) TRMM and the (right) GMMF RPFs in three size categories: (first row) small, (second row) medium to large, and (third row) very large. The Level-2 TRMM RPF data is mapped to the GEOS 2.0° x 2.5° (lat-lon) grid boxes; relative percentages sum to 100%. TRMM orbital sampling biases have been removed by using the reciprocal of the total number of observed pixels in each box as weighting.


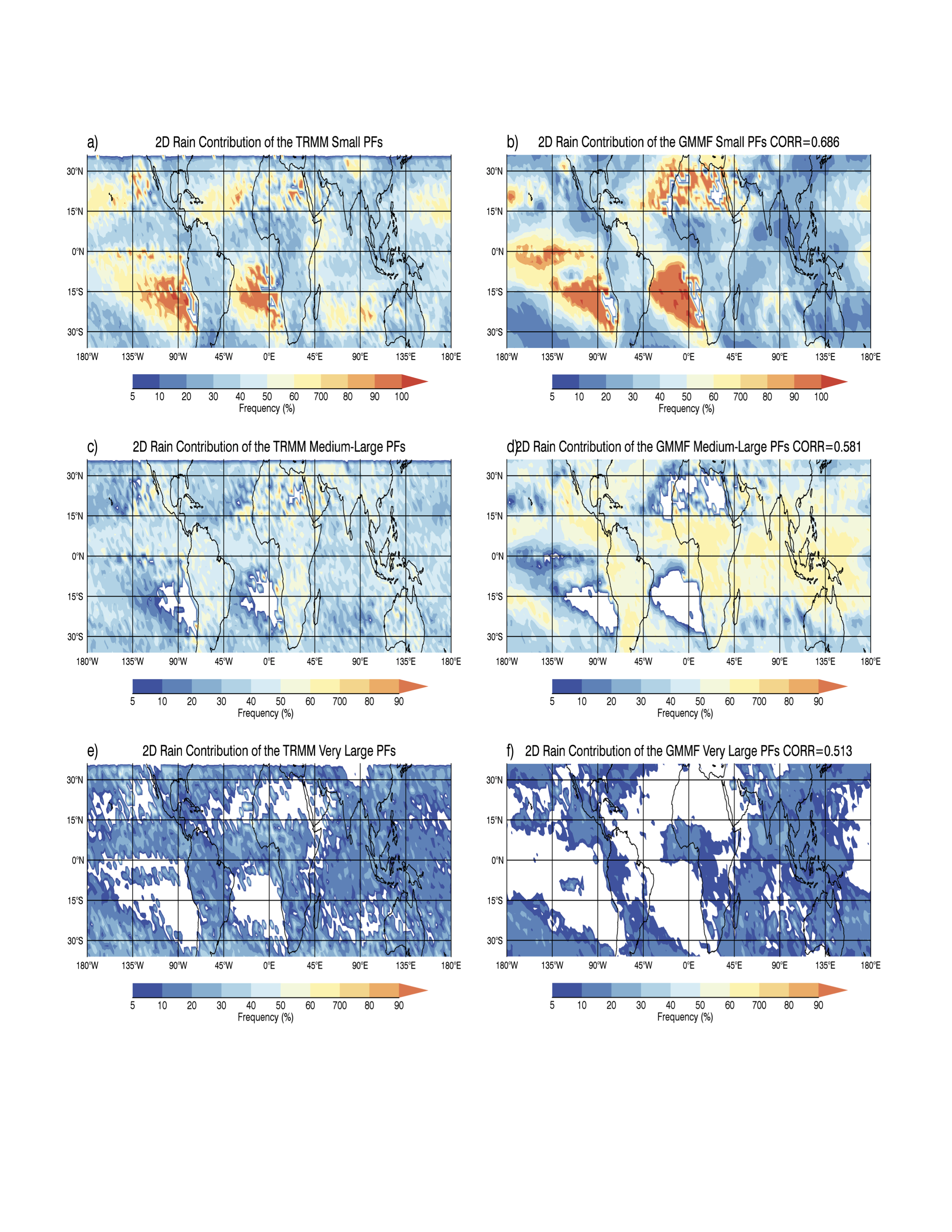


**Figure S2.** Annual two-dimensional (2D) local rainfall contribution at each GEOS grid box from the (left) TRMM and the (right) GMMF RPFs in three size categories: (first row) small, (second row) medium to large, and (third row) very large.


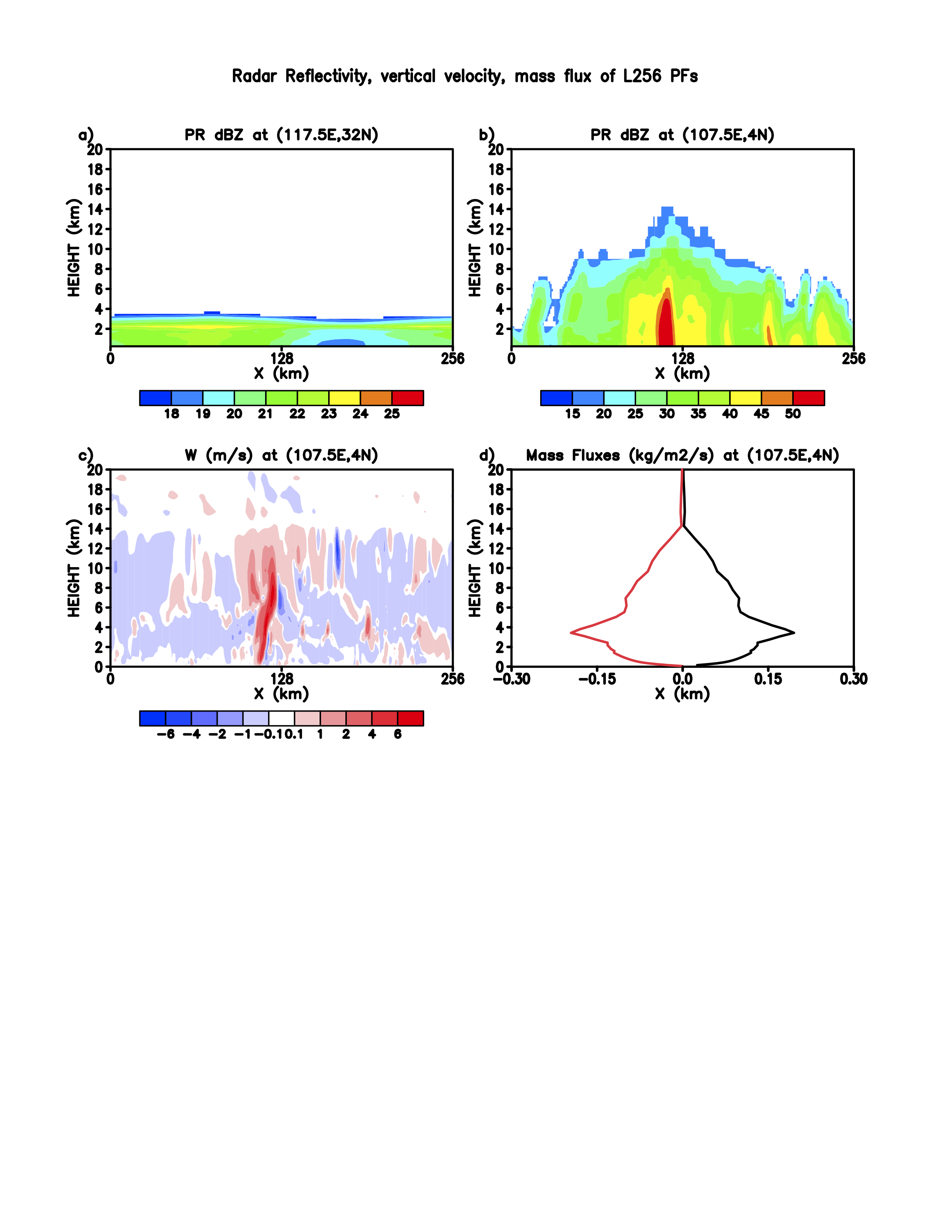


**Figure S3.** Instantaneous radar reflectivity (dBZ) of 256km-wide precipitation features from the embedded 2D GCE at GEOS grid a) (117.5E, 32N) and b) (107.5E, 4N) at 0000 UTC 3 January 2007. c) Same as b) except for the vertical velocity (m/s). d) Domain average upward (black) and downward (red) mass flux (kg m^-2^ s^-1^) from Figure S3c.
